# A Review of Phytochemicals and Bioactive Properties in the Proteaceae Family: A Promising Source of Functional Food

**DOI:** 10.3390/antiox12111952

**Published:** 2023-11-01

**Authors:** Jiale Zhang, Michael E. Netzel, Andrew Pengelly, Dharini Sivakumar, Yasmina Sultanbawa

**Affiliations:** 1 ARC Industrial Transformation Training Centre for Uniquely Australian Foods, Queensland Alliance for Agriculture and Food Innovation (QAAFI), The University of Queensland, Indooroopilly, QLD 4068, Australia; jiale.zhang@uq.edu.au (J.Z.); m.netzel@uq.edu.au (M.E.N.); d.sivakumar@uq.edu.au (D.S.); 2 Indigenous Plants for Health Association, 196 Bridge St, Muswellbrook, NSW 2333, Australia; trueunicorn11@gmail.com; 3 Phytochemical Food Network, Department of Crop Sciences, Tshwane University of Technology, Pretoria 0001, South Africa

**Keywords:** Proteaceae, indigenous fruits, phytochemicals, bioactive properties, functional ingredients

## Abstract

In recent decades, natural plant-based foods have been increasingly used to improve human health due to unhealthy modern dietary patterns, such as the consumption of foods high in sugar and fat. Many indigenous species have been used by Aboriginal peoples for their food and therapeutic properties. Thus, it is important to understand the health-enhancing bioactive profile of Australian indigenous species. The Proteaceae family, such as the genera of *Protea*, *Macadamia*, and *Grevillea*, have been commercially used in the horticulture and food industries. Researchers have reported some findings about *Persoonia* species, one of the genera in the Proteaceae family. The aim of this review was to provide an overview of the family Proteaceae and the genus *Persoonia*, including distribution, traditional and commercial uses, phytochemicals, bioactive properties, potential opportunities, and challenges. In this review, bioactive compounds and their properties related to the health benefits of the Proteaceae family, particularly the *Persoonia* genus, were reviewed for potential applications in the food industry.

## 1. Introduction

The use of traditional food has been an important trade for thousands of years and the value addition to edible plants harvested from the land of Australian Aboriginal communities is one of the potential enterprise opportunities [1,2]. Due to urbanization and environmental challenges, traditional food has been used nationally and internationally for food security and diet diversity [3]. Modern dietary patterns (or “Western diets”) are often highly processed, high in fat, unsaturated fatty acids, and sugar, and low in essential micronutrients and fibre, and contribute to several diet-related diseases, such as coronary heart disease, high blood pressure, and diabetes. Whereas traditional (plant-based) foods are usually high in protein, fibre, and essential micronutrients [4]. Thus, natural plant-based products have positive impacts and become very vital to human health [5]. For example, the high vitamin C content of the Kakadu plum (*Terminalia ferdinandiana*), the bush tomato (*Solanum centrale*) with high carbohydrate levels, Davidson’s plum (*Davidsonia pruriens*) with a high vitamin A content, and the quandong (*Santalum acuminatum*) with high folates, are four of the most popular native fruits commercialized in the Australian market [6].

The Proteaceae family, which occurred in Gondwana over 100 million years ago [7], one of the largest flowering plant families, comprises more than 80 genera and 1800 species recorded in the world [8]. The Proteaceae family is predominantly distributed in the southern hemisphere and approximately 45 genera and 1100 species are diverse in Australia, while 37 genera are native to Australia [9]. The name Proteaceae originated from the Greek sea god, Proteus, and was named by Carl Linnaeus in 1767 [10]. The first monograph on the family Proteaceae was published by Robert Brown in 1810 and it was classified into two subfamilies based on the 38 genera found by Weston in 1995 [11]. Then, Peter Weston finally revised the subfamilies into five groups: *Persoonioideae* (5 genera), *Bellendenoideae* (1 genus), *Grevilleoideae* (47 genera), *Proteoideae* (25 genera), *Symphionematoideae* (2 genera) [12]. Plants from the Proteaceae family are shrubs or trees from 0.2 to 40 m in height with a variety of leaves, often leathery, rarely fleshy, spinescent, or toothed. The venation of the plants is usually brochidodromous, pinnate, palmate, or parallel [7]. The plants usually are bisexual, whose flower parts are actinomorphic to strongly zygomorphic. Proteaceae plants in many taxa are self-pollinated flowers, contributing to a low flower-to-fruit ratio due to inbreeding depression. In particular, a few plants are sterile and have natural vegetative propagation, such as *Lomatia tasmanica* and *Hakea pulvinifera*. Inflorescences may be compound or simple, often forming racemes. Fruits of the family Proteaceae are diversified into the dehiscent or leathery follicle, indehiscent drupe or falsely drupe, and developed by solitary carpel [7].

The *Persoonia* genus, belonging to the family Proteaceae, comprises about 100 species that are endemic to Australia [13]. *Persoonia* spp. are commonly described as Geebung, which originated from the Aboriginal term ‘jibbong’ [14]. They are insect-pollinated shrubs or small trees ranging from 0.2 to 25 m in height [15,16]. *Persoonia* spp. have diverse leaves including fine, round, elliptical, lanceolate, linear, grass-like, finely ribbed, pungent, and pine-like [15]. Anthers and tepals vary in colour from bright or greenish yellow to white with auxotelic or anauxotelic inflorescences [15]. The fruit is green, purple, or black in colour [15]. The flowering and fruiting are continuous throughout the year but with poor propagation by human attempts (as low as 0.01% in terms of fruit-to-flower ratio) due to the ability of complex dormancy mechanisms [15,16,17]. *Persoonia* spp. are prostrate or shrubs to small trees with light green single leaves, smooth bark and yellow flowers arranged singly or in a raceme, which is a drupe, up to 0.1–25 m high [18]. Most species are grown in fire-prone areas of eastern Australia, which contributes to the poor propagation success of *Persoonia* spp. [16]. Rare *Persoonia* spp. seem to receive few pollinators compared with common species [19]. Thus, 15 *Persoonia* species have been conserved and documented as vulnerable, critically endangered, or extinct under the Biodiversity Conservation Act of 2016 and the Environment Protection and Biodiversity Conservation Act of 1999 (EPBC Act) [20] and all of them are distributed in the NSW region [19,21].

*Persoonia* spp. is one of the popular food sources consumed by Australian Aboriginal people. The fruit, seed, and kernel are edible [22,23]. *Persoonia* spp. has shown potential applications in bactericidal treatment for a range of maladies [24,25,26,27]. A few studies have reported antimicrobial activities in the genus *Persoonia*. *Persoonia falcata* contained saponins and tannins used in the treatment of gastrointestinal disorders, colds/flu and eye disorders [28]. *P*. *juniperina* and *P*. *pinifolia* exhibited high inhibitory effects against Gram-positive and Gram-negative bacteria, respectively [25]. Therefore, the genus *Persoonia* is one of the traditional plants that have potential applications in the nutraceutical or food industry. 

Comprehensively understanding the potential values of indigenous Australian plants is important for people to develop functional food products. “Functional foods” are foods that have unique properties that provide additional physiological benefits [29]. However, the genus *Persoonia* has been less reviewed and reported on in the literature. Therefore, *Persoonia* spp. should be studied as a native plant of interest. This can provide a better understanding of its bioactive properties and potential health benefits for the Australian Aboriginal communities, as well as for the functional food industry and can improve food security. This review aimed to provide knowledge on the distribution, traditional and commercial use, phytochemicals (including their distribution on the plant), and bioactive properties of the Proteaceae family and the genus *Persoonia*.

## 2. Methodology

To evaluate the distribution, traditional and commercial use, phytochemicals (including their distribution on the plant), and bioactive properties of the Proteaceae family and the genus *Persoonia*, this review compiled information from the literature and was conducted utilizing several electronic databases including the Web of Science, PubMed, Wiley online library and Google Scholar databases. Search terms such as bioactive, biological activities, bioactivities, bioactive properties, phytochemistry, *Persoonia*, and Proteaceae, etc. were used to perform the literature search.

## 3. Distribution

Proteaceae is a large family with a wide distribution. It started on the supercontinent of Gondwana and this family diversified successfully before the fragmentation of Gondwana [7]. There is evidence of fossil pollen history found in Gondwana regarding the origination of Proteaceae [7,30]. A large amount of proteaceous pollen was found in the Cretaceous coalmine of the South Island of New Zealand [30]. The family Proteaceae is most diverse in Australia, Africa, and South America, followed by Central America, Asia, India, Fiji, Indonesia, Japan, New Zealand, New Caledonia, Sri Lanka, Vanuatu, Micronesia, Madagascar, Solomon Islands, and Papua New Guinea [31]. The subfamily *Persoonioideae* is only distributed in Australia, New Zealand, and New Caledonia including five genera, of which *Persoonia*, *Acidonia*, and *Placospermum* are in Australia, one (*Garnieria*) in New Caledonia, and one (*Toronia*) in New Zealand [15,32].

The *Persoonia* genus is mainly distributed in the subtropical to temperate regions of northern, south-eastern, and southwestern Australia [16]. The genus *Persoonia* shows great diversity in southwestern and southeastern Australia. The majority of *Persoonia* species are grown in sclerophyll woodlands and shrublands, while few are in rainforests [33]. Except for *P*. *pertinax*, it is only distributed in the Great Victoria Desert region of Western Australia [15]. There are 99 species grown in Australia, of which 49 are native to New South Wales, 43 to Western Australia, 19 to Queensland, 11 to Victoria, 4 to Tasmania, 1 to the Northern Territory, and 1 to Southern Australia, respectively. *P*. *linearis* distributed in southeastern Australia is the most abundant species in the genus *Persoonia*, followed by *P*. *levis*, *P*. *juniperina*, *P*. *falcata*, *P*. *glaucescens*, *P*. *confertiflora*, *P*. *nutans*, and *P*. *pinifolia* [34]. The distribution of *P*. *falcata* is the broadest across over 3000 km in the regions of northern Australia. This could be due to less rainfall in the southern regions, which leads to a difficulty in propagation and growth [32].

## 4. Traditional and Commercial Use

The family Proteaceae has provided potential economic value and contributed to a range of products in the food industry, pharmaceutical industry, horticultural industry, and material industry. Table 1 summarizes the traditional and commercial use of the family Proteaceae. 

The fruits, seeds, nuts, and flowers of several Proteaceae species have provided food security. In particular, the fact that over 50,000 tons of *Macadamia* nuts from Australia were produced and exported in 2020 plays an important role in the global nut industry [35]. This is a 1.5-fold increase over the corresponding value for 2014 and a 30-fold increase compared with the value in 1987 [11]. Nevertheless, only *Macadamia integrifolia* and *Macadamia tetraphylla* can be edible as food to provide nutrients and potential pharmacological chemicals, while *Macadamia jansenii* and *Macadamia ternifolia* contain the toxic cyanogenic glycosides, which cannot be consumed by people [11,36,37]. Proteaceae species also developed many medicinal applications and have traditionally been used by Aboriginal people, such as for eye infections, sore throats, skin infections, gastroenteritis, respiratory infections, kidney problems, liver diseases, and inflammation treatments (Table 1). For example, the leaves and flowers of *Oreocallis grandiflora* contain flavonoids and have the potential to be used in anti-inflammatory and diabetes treatments [38]. Many Proteaceae species can treat skin infections or be used as skin-lightening products as listed in Table 1. However, some species are harmful to people. For instance, people who have an allergy to resorcinol cannot directly be in contact with the flowers or trees of the genus *Greville*, which could lead to allergic contact dermatitis [39]. Cultivation is another commercial use in the family Proteaceae. *Persoonia longifolia* has been used in cultivation in the UK since 1850 but rarely in Australia [40]. *Protea*, *Leucospermum*, and *Leucadendron* are the three major cultivars in the Proteaceae family, whilst other Proteaceae species also have applicability for ornamental uses. For instance, the genus *Banksia* has been commercialized as cut and dried flowers and the genus *Grevillea* can be used as a landscape plant due to its characteristic of drought tolerance [41]. As most Proteaceae species are shrubs or trees, they are a good source of timber, contributing to the materials industry. For example, *Grevillea* and *Hakea* are used in the manufacture of boomerangs [42].

The genus *Persoonia* has been commonly used as food and medicine in Australian Aboriginal communities. For example, *P*. *falcata* found in the Madjedbebe region was consumed as a plant-based food 65,000–53,000 years ago and it is still a highly sought-after fruit for the local Aboriginal people [43]. There are numerous reports that *Persoonia* species are edible, especially the fruits on the ground that are the best and softest, tasting like nibbling sweet candy floss, including *P*. *pinifolia*, *P*. *linearis*, and *P*. *levis* [44,45,46,47]. Aboriginal peoples from New South Wales normally discard the skin and eat the pulp [45]. Some Aboriginal peoples eat the whole fruit without the seed, depending on their lifestyle. However, eating the whole fruit has benefits for human health because different parts of the fruit have different bioactive compounds, nutrients, and metabolites [48], which need to be further studied to prove the relationship between them. *P*. *levis* was described as the most popular ‘Indigenous bush lollies’ after they have ripened [46]. *P*. *virgata* also has the potential to be an ornamental crop [44]. The leaves and wood of *P*. *falcata* have been reported as a plant-based medicine used for sore eyes, diarrhea, and chest infections due to the presence of saponins and tannins [49,50]. Moreover, some *Persoonia* spp. used as a protection infusion can prevent the fraying of string and fishing lines by using the bark of plants, like *P*. *linearis* and *P*. *laurina* [51,52].

**Table 1:** Summary of usage of the family Proteaceae.

Industry	Genus/Specie	Traditional/Commercial Use	References
Food industry	*Persoonia*, *Hicksbeachia*, *Floydia*, *Macadamia*, *Hakea*, *Brabejum*, *Finschia*, *Gevuina*, *Panopsis*, *Oreocallis*	Seeds, nuts, gum, or fruits have been eaten by Australian Aboriginal people	[7,11]
Food industry Pharmaceutical industry	*Helicia serrata*, *H*. *robusta*	Young shoots eaten by Javanese people	[7]
	*Telopea*, *Lambertia*, *Grevillea*, *Banksia*, *Macadamia*	Honey sources	[11,53,54]
	*Hakea leucoptera*	The roots are used for freshwater	[55]
	*Banksia*, *Persoonia*	Relief of coughs and sore throats	[54,56]
Pharmaceutical industry Horticultural industry	*Dilobeia thouarsii*, *D*. *cordata*	Leaves against *S*. *aureus* used for skin infection in Madagascar	[57]
	*Lomatia hirsuta*	Leaves used for the treatment of bronchitis and asthma in Chilean traditional medicine	[58]
	*Faurea saligna*	Diarrhea	[7,56,59]
	*Grevillea*, *Hakea*, *Persoonia*, *Roupala* and *Xylomelum*	Skin infections	
	*Heliciopsis*	Eye infections	
	*Protea*	Skin infections or hyperpigmentation	[60,61]
	*Helicia robusta*	Gastritis or kidney problems	[62,63]
	*Oreocallis*	Liver diseases, bleeding, or inflammation treatments	[7,38]
	*Helicia*, *Grevillea*	Skin or mouth sores	[56,64]
	*Grevillea*, *Hakea*, *Persoonia*, *Roupala*, *Xylomelum*, *Aulax*, *Leucadendron*, *Paranomus*, *Leucospermum*, *Mimetes*, *Heliciopsis*, *Toronia*, *Banksia*	Skin lightening agent	[7,59,65]
	*Leucospermum*, *Persoonia*, *Hakea*, *Grevillea*, *Protea*, *Serruria*, *Waratah*, *Banksia*, *Telopea*, *Isopogon*, *Leucadendron*	Colourful horticultural plant	[41,55,66,67]
Material industry	*Persoonia*	Fishing lines and strings	[11]
	*Grevillea*	Cementing compound	[55]
	*Grevillea*, *Protea*, *Darlingia*, *Buckinghamia*, *Athertonia* and *Hakea*	Timber	[7,11,42]

## 5. Phytochemicals

Phytochemical studies of natural plants are providing a pathway to drug discovery, which is essential for medical needs and discovering potential therapeutic values for human health. Plants can produce two types of organic compounds: primary and secondary metabolites, of which secondary metabolites are intermediate or produced in simulation pathways of stress response derived from primary metabolites [68]. Although the mechanisms of secondary metabolites have not been fully understood, an increased scientific interest in phytochemicals was demonstrated in the past two decades [69]. Phytochemical studies are helpful in the research and development of potential drugs or products, such as pharmaceuticals (drugs, poisons, or stimulants), food (dairy products, additives, colours, or spices), horticultural (pesticides or nutrient additions), and manufacturing industries (fuels or colouring additives). Generally, phytochemicals are classified into six main types including carbohydrates, lipids, alkaloids, quinones, phenolic compounds, and terpenoids [70], which not only play important roles in plant growth but also provide benefits for human beings.

Currently, only 30% of Proteaceae species have been investigated for phytochemical profiling, of which less than 10% of species have had their compounds isolated and purified. The *Grevillea* and *Protea* genera have the widest use and study [71] with 362 species in *Grevillea* and 112 species in *Protea* described in previous studies [11]. There are fewer than 400 compounds identified in the family Proteaceae, including three main categories: phenolic compounds (69%), quinones (8%), and alkaloids (13%) [71,72]. The Proteaceae species have high potential bioactive properties attributed to the high proportion of phenols and polyphenols, including antioxidant, anti-inflammatory, antibacterial, antiallergic, anticancer, and antiviral activities. Phenolic glucosides, alkylresorcinols, and their derivatives, and tropane alkaloids are the three biggest phytochemical groups found in the family Proteaceae [71].

Only the novel discovered phytochemicals in the previous studies were listed until 2023, except for compounds (15, 36–39: Icariside B_1_, Kaur-16-ene, and Farnesylacetone) that were not included in the previously reviewed studies [71,72]. There are 39 novel compounds first identified in *Protea cynaroides*, *Grevillea robusta*, *Heliciopsis terminalis*, *Stenocarpus sinuatus*, *M*. *integrifolia*, and *Roupala montana*. The majority of them are phenolic compounds. Compounds 1–4 (3,4-bis(4-hydroxybenzoyl)-1,5-anhydro-d-glucitol, 4-hydroxybenzoyl-1,5-anhydro-d-glucitol, 2-(hydroxymethyl)-4-oxo-4H-pyran-3-yl-6-*O*-benzoate-β-d-glucopyranoside, and 3-hydroxy-7,8-dihydro-β-ionone-3-*O*-β-d-glucopyranoside) identified in the leaves of *P*. *cynaroides* exhibited weak inhibitory activity against the tyrosinase enzyme and can be used as an inhibition agent for the reduction of melanin pigments, but compounds 5–6 (3,4-dihydroxybenzoic acid and 3-hydroxykojic acid) have inhibitory activity with the IC_50_ value of 149.2 ± 1.06 and 274.5 ± 2.12 µg/mL, respectively [57]. The stable activity of tyrosinase inhibition could be due to free 3-OH or 5-OH groups found in both compound 3 (2-(hydroxymethyl)-4-oxo-4*H*-pyran-3-yl-6-*O*-benzoate-β-d-glucopyranoside) and kojic acid [57]. Some bioactive properties have been found in compounds 7–8, 10–12, and 14 (B-type Procyanidin, Diosmetin, 6-hydroxy coumarin, *p*-hydroxybenzeldahyde, Methyl gallate, and Ethyl gallate) including antimicrobial activity (compounds 7, 8, 12, and 14: B-type Procyanidin, Diosmetin, Methyl gallate, and Ethyl gallate), antioxidant activity (compounds 8, 14: Diosmetin and Ethyl gallate), anticancer capacity (compound 10: 6-hydroxy coumarin), and antimalarial activity (compound 11: *p*-hydroxybenzeldahyde) [73,74,75,76,77]. Compound 15 (Icariside B_1_) is a good inhibitor of breast cancer against estrogen receptor alpha [78]. Compounds 16–23 (Heliciopside A, Heliciopside B, Heliciopside C, Heliciopside D, Heliciopside E, Clemochinenoside D, 3,4,5-trimethoxyphenyl-β-d-glucopyranoside, and Kusukuenol B_1_) have been investigated for antidiabetic activity, of which compounds 18 and 20 (Heliciopside C, and Heliciopside E) have the highest potential stimulatory effects for type 2 diabetes mellitus [79]. Compound 24 (Ursolic acid) extracted from the trunk of *H*. *terminalis* has an anti-elastase activity (IC_50_ = 34.3 ± 0.6 µmol/L) [80]. Compounds 25–35 showed potential antiaging activity when applied in skin care or skin-whitening products [81,82]. Generally, most phytochemicals were identified in the leaves, followed by barks, fruits, flowers, and roots in the Proteaceae family. Only 10% of the phytochemical studies investigated the fruits of the Proteaceae family, contributing to a gap in understanding the constituents of Proteaceae plants. Thus, the family still has huge potential in applications to meet human needs and for the exploration of more useful plants to reduce food insecurity or even climate change [83].

A summary of the phytochemicals of the family Proteaceae was presented in Table 2. 

## 6. Bioactive Properties

Fruits are an important source of nutrients and dietary energy, providing fibre, minerals, vitamins, and phytochemicals, and have been consumed for their nutritional and health value. Many fruits have proven their potential protective effects against different types of diseases, such as cardiovascular diseases, cancers, eye diseases, chronic diseases, and obesity [87,88]. Currently, over 25,000 plants have been found in Australia, of which around 2000 edible plants have been consumed by people and some exported worldwide [89]. Australian native fruits are seasonal and distributed widely in arid and non-arid regions of Australia [6], which can satisfy the needs of customers throughout the year. Numerous indigenous fruits have emerged in the Australian market and made an economic contribution, such as bush tomato, Davidson’s plum, Kakadu plum, lemon aspen, pepper berries, quandong, and riberry. The Australian native food industry continues to develop slowly due to the challenges to commercialize and meet market demands both domestic and international [90]. However, these plants constitute a promising source of edible fruits with bioactive properties, such as anti-inflammatory, antidiabetic, antioxidant, and antimicrobial activities [91].

Plant-based products have been widely accepted and commercialized. This is because more and more active compounds from natural sources have been found to be beneficial for human health. These compounds normally contain a series of properties to prevent the invasion of pathogens and bacteria from plants, contributing to the health benefits in the human body as well. Numerous known and novel compounds were discovered from the family Proteaceae with potential bioactive values. Five main bioactive properties of the family Proteaceae have been summarized including antioxidant activity, antimicrobial activity, cytotoxicity, anti-inflammatory, and antiviral activity—only quantitative studies are listed in Table 3, Table 4, Table 5 and Table 6. Generally, the leaves have been a frequently used study material among four bioactive assays, followed by barks, stems, flowers, and other tissues of the plants.

### 6.1. Antioxidant Activity

**Figure 1:**
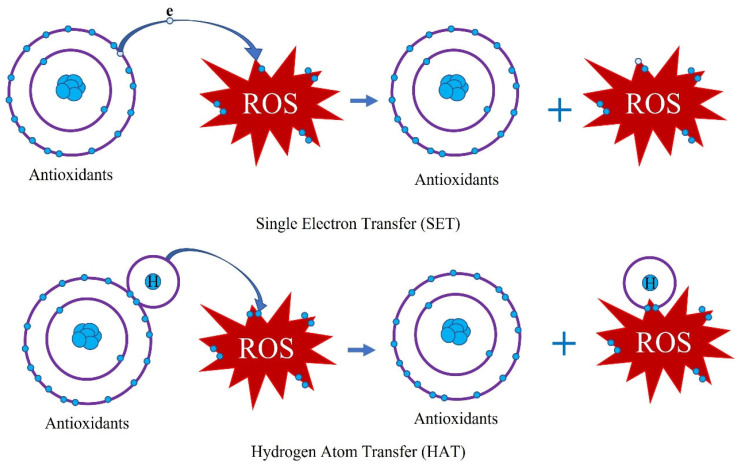
The mechanism of hydrogen atom transfer (HAT) and single electron transfer (SET) assays.

The fact that unbalances between free radicals and antioxidants leads to oxidative stress in the human body possibly causes respiratory diseases, cancers, aging, and multiple disorders [92]. Many free radicals produced from the metabolizing oxygen of cells can lead to this unbalance, including hydroxyl, superoxide, nitric oxide, hydroperoxyl, nitrogen dioxide, and lipid peroxyl radicals [93]. However, the uptake of antioxidants can reduce the presence of free radicals to prevent diseases. Thus, the determination of the antioxidant activity in plants and derived (food) products is useful. The methods of antioxidant activity have been remarkably developed in recent decades. Except for chromatographical and electrochemical methods, the determination of antioxidant activity is mainly divided into two categories using spectrometry: hydrogen atom transfer (HAT) and single electron transfer (SET) assays (Figure 1). The mechanism of HAT is to measure the ability of hydrogen donation of antioxidants transferred to free radicals, while the mechanism of SET is to determine the capacity of antioxidants to reduce metals, carbonyls, and radicals by donation of an electron [93]. Typical methods of HAT assays are the oxygen radical absorbance capacity (ORAC), the total peroxyl radical-trapping antioxidant parameter (TRAP), and the total oxyradical scavenging capacity (TOSC) assays, whereas the common examples of SET assays include the total phenolic content (TPC) using Folin–Ciocalteu reagent, the ferric reducing antioxidant power (FRAP), and the cupric antioxidant capacity (CUPRACA) [92]. Moreover, the 2,2-diphenyl-1-picrylhydrazyl (DPPH) radical scavenging capacity assay and the Trolox equivalent antioxidant capacity (TEAC) are two combined mechanisms of SET and HAT [92]. Currently, TPC, FRAP, and DPPH are widely used to measure the antioxidant activity of plants, although these methods cannot detect lipophilic compounds [92,93]. According to Chaves’s study, it is recommended that at least two different methods should be considered to measure antioxidant activity during the study [94].

Most studies on the antioxidant activity of Proteaceae species used TPC, FRAP, and DPPH, rather than ORAC and TEAC, as with a few publications (Table 3). This could be because TPC, DPPH, and FRAP assays are simple to run, cost-effective and rapid, although these methods are unable to analyse both hydrophilic and lipophilic compounds. According to Table 3, a wide range of studied plant materials has been reviewed from shoot system to root system, which means the Proteaceae species have potential bioactive values on each part of the plant. It has also been found that some studies focused on the same species, and generally presented varying results, which could be attributed to the choice of extraction solvents, plant locations, and extraction methods. For example, the aqueous ethanol extract of *O*. *grandiflora* leaves collected from Ecuador, South America, has a radical scavenging capacity with an IC_50_ value of 6.69 ± 1.39 μg/mL, while an IC_50_ value of 292.37 ± 9.37 µg/mL was analysed in the absolute ethanol extract of *O*. *grandiflora* leaves collected from Ecuador, South America [38,95]. Thus, the extraction solvent has a crucial impact on the quantification analysis, which could be attributed to the solubility of these chemicals. The different methods used are another factor that influences the results. *O*. *grandiflora* flowers collected from the same location but using the different procedure of a DPPH assay exhibited different values of radical scavenging activity at IC_50_ values of 14.39 ± 1.43 μg/mL and 955.23 ± 0.25 µg/mL, respectively [38,96]. *Roupala paulensis* (aerial parts) has the highest TP content (24.27 ± 0.76 g GAE/100 g) compared to other Proteaceae species, but the DPPH value of *R*. *paulensis* was not promising, which could be due to the sensitivity of the DPPH radical scavenging capacity assay, such as Lewis bases, light, oxygen, and solvent types [97]. This contributes to the influence of quantitative analysis. *H*. *terminalis* was the only species focusing on the trunk and it showed a promising antioxidant value (IC_50_: 156.9 mg/mL) using the DPPH assay [98]. However, measuring other antioxidant methods is necessary to provide a more comprehensive result.

**Table 3:** Summary of the antioxidant activity of the family Proteaceae.

Species	TP	FRAP	DPPH (IC_50_)	ORAC	TEAC	References
*G*. *avellana* (nut)	1.9–4.6 g GAE/100 g	51.2–352.8 mM TE/g	8.9–93.8% inhibition at 100 µg/mL	273.9–2157.5 μM TE/g	207.3–1012.8 μM TE/g	[99]
*Macadamia* (nut)	-	-	-	14.43 ± 2.31 μM TE/g	-	[100]
*M*. *integrifolia* (nut)	52.9–108.6 µg GAE/g	4.7–51.9 µM Fe^2+^/g	0–57.0% inhibition (without conc.)	-	13.3–118.8 mg TE/g	[101]
*H*. *terminalis* (trunk)	-	-	156.9 mg/mL	-	-	[98]
*Faurea*. *Speciosa* (leaf)	65.4 ± 0.5 mg AAE/g	-	499.4 ± 5.8 μg/mL	-	-	[102]
*Protea Susara* (aerial part)	-	4.4 ± 0.1 μM Fe^2+^/g	41 ± 2% inhibition at 0.5 mg/mL	-	-	[103]
*B*. *menziesii* (floral)	26.1 ± 4.1 mg GAE/100 g	2.90 ± 0.55 mM Fe^2+^/kg	1095 ± 497 µM TE/kg	-	-	[104]
*B*. *sessilis* (floral)	31.8 ± 5.5 mg GAE/100 g	3.12 ± 0.61 mM Fe^2+^/kg	1093 ± 263 µM TE/kg	-	-	
*M*. *tetraphylla* (peel)	168.22 ± 0.77 mg GAE/g	1607.82 ± 7.89 μM TE/g	1128.76 μM TE/g	-	-	[105]
*O*. *grandiflora* (flower)	-	-	14.39 ± 1.43 μg/mL	-	-	[38]
*O*. *grandiflora* (leaf)	-	-	6.69 ± 1.39 μg/mL	-	-	
*Roupala paulensis* (aerial parts)	24.27 ± 0.76 g GAE/100 g	-	37.50 ± 0.46 μg/mL	-	-	[106]
*Adenanthos sericeus* (stem)	-	-	57.3–82.8 μg/mL	-	-	[107]
*H*. *sericea* (fruit)	186.3 mg GAE/g	3.4 mM Fe^2+^/g	11.6 µg/mL	-	-	[108]
*O*. *grandiflora* (leaf)	13.97 ± 0.31 GAE mg/100 g	-	292.37 ± 9.37 µg/mL	-	-	[95]
*F*. *saligna* (leaf)	-	-	1.17 ± 0.04 µg/mL	-	-	[109]
*F*. *saligna* (stem and bark)	-	-	13 ± 1 µg/mL	-	-	[110]
*O*. *grandiflora* (flower)	-	-	955.23 ± 0.25 µg/mL	-	-	[96]
*Embothrium coccineum* (leaf)	-	0.40–0.53 mM Fe^2+^/g	5.27–21.78 mg/mL	270.61–405.21 μM TE/g	-	[111]
*H*. *sericea* (stem)	267.6 ± 5.9 mg GAE/g	-	9.5 ± 0.1 mg/L	-	-	[112]
*H*. *sericea* (leaf)	217.0 ± 2.7 mg GAE/g	-	13.4 ± 0.4 mg/L	-	-	
*H*. *sericea* (fruit)	110.1 ± 2.7 mg GAE/g	-	28.3 ± 1.8 mg/L	-	-	

**Figure 2:**
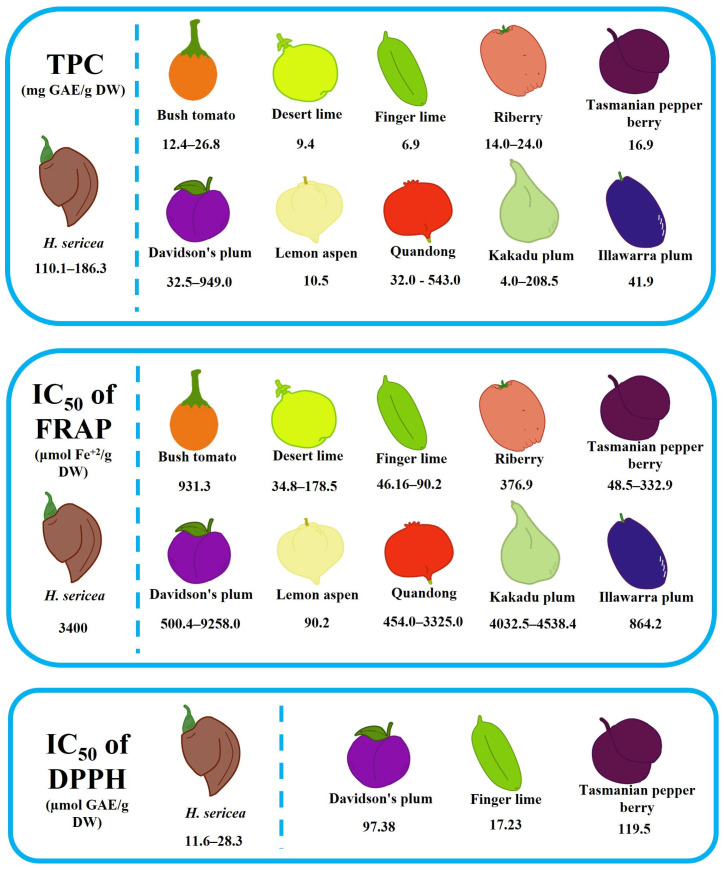
Comparison of the antioxidant activity of Proteaceae species and Australian native fruits in different assays [6,108,112,113,115,119,120,121,122,123,124,125].

Currently, only three Proteaceae fruits have been studied for their antioxidant and antimicrobial activities: *H*. *sericea*, *H*. *salicifolia*, and *P*. *linearis*. The fruit of *H*. *sericea* is the only species in the Proteaceae family studied for its antioxidant activity [108,112]. Figure 2 shows the antioxidant activity of the fruits of *H*. *sericea* and Australian native fruits. Ellagic acid is the main compound found in the fruit of *H*. *sericea* [112]. Compared with other Australian native species, the ellagic acid content in the fruit of *H*. *sericea* (3700 ± 60 mg/100 g DW) is higher than Davidson’s plum (15–3640 mg/100 g DW), Kakadu plum (8–880 mg/100 g DW), quandong (9 mg/100 g DW), and muntries (16 mg/100 g DW) [113,114,115,116,117,118]. The TPC and FRAP in the fruit of *H*. *sericea* are higher than that of bush tomato, desert lime, finger lime, riberry, pepper berry, lemon aspen, and Illawarra plum, respectively (Figure 2). Thus, the ellagic acid content is consistent with the antioxidant activity in the fruits and it could be the major contributor to its antioxidant properties. However, the fruit of *H*. *sericea* in DPPH radical scavenging activity is lower than that of pepper berry, which could be mainly because pepper berry was collected from different locations: Brisbane [119] and Tasmania [120]. It proved that the fruits from different locations with variable growth conditions contribute to the difference in functional and nutritional values. Moreover, there is a variation in the antioxidant activity of some Australian native species (Figure 2). This could be attributed to different sample locations, sample extractions, and sample growth conditions.

### 6.2. Antimicrobial Activity

Foodborne microorganisms are harmful to human health, which could lead to some illnesses. There are several predominant foodborne pathogens listed by the New South Wales Food Authority including *Bacillus*, *Salmonella*, *Campylobacter*, *Escherichia*, *Staphylococcus*, and *Listeria* spp. [126]. For example, people who are infected with *Staphylococcus aureus* could have symptoms of vomiting and stomach cramps within 0.5 to 8 h. Furthermore, the discovery of antibiotics contributes to the treatment of human illnesses, but it also leads to the drug resistance of microorganisms. Therefore, the discovery of new antibiotic compounds is an important objective for future medicines. Many studies have illustrated that natural sources provide many compounds with potential antimicrobial properties, such as *Terminalia carpentariae* [127], *Terminalia ferdinandiana* [128], *Acacia floribunda* [129], *Macadamia integriflora* [130], and *Hakea sericea* [131]. Currently, there are several standardized methods to analyse antimicrobial activity including the diffusion assay, dilution assay, bioautography, time-kill test, ATP bioluminescence assay, and flow cytofluorometric method, of which the diffusion and dilution assays are the most common methods [132]. This is mainly because the methods other than the diffusion and dilution assays require special techniques and further complex statistical analysis. Agar disk and the well diffusion method are the official and approved standards to test antimicrobial susceptibility, although they have no ability to distinguish bactericidal and bacteriostatic impacts [132]. Therefore, dilution methods, as the quantitative measurement, can be used to determine the minimum concentration of antimicrobial drugs to visibly inhibit the growth of microorganisms, which is also called the minimum inhibitory concentration (MIC). The minimum bactericidal and fungicidal concentration (MBC/MFC) can determine the minimum concentration of antimicrobial agents that kill 99% of microorganisms. To enhance the accuracy of visible results, some dye reagents, as the indicator, have been researched, such as 2,3,5-triphenyltetrazolium chloride (TTC) [133], resazurin [134], and 3-[4,5-dimethylthiazol-2-yl]-2,5-diphenyltetrazolium bromide (MTT) [132].

*Protea*, *Grevillea*, and *Hakea* are three popular interest genera in bioactive studies of the Proteaceae family. Antimicrobial activity was the highest quantified assay in the Proteaceae plant studies compared to others. Five microbes are mostly studied to assess the antimicrobial activity of the family Proteaceae: *E*. *coli* (20 studies), *S*. *aureus* (19), *P*. *aeruginosa* (11), *C*. *albicans* (9), and *B*. *cereus* (9). The fruit of *M*. *integrifolia* has been studied against *E*. *coli* with the highest microbial activity at a MIC value of 5.3 µg/mL [130], compared to >100 μg/mL MIC value in *Roupala brasiliensis* stem [135], 31.125 ± 0.2 μg/mL in leaves of *Embothrium coccineum* [136], 156 μg/mL in *Darlingia darlingiana* bark [137], and other species. In *S*. *aureus* studies, *Roupala brasiliensis* stem has the highest antimicrobial activity at a MIC value of 15.6 μg/mL [138], whilst aerial tissues of *G*. *avellana* showed antibacterial activity against *P*. *aeruginosa* with a MIC value of 64 µg/mL [139]. *M*. *integriflora* (leaf) exhibited the highest MIC values at 6.5 μg/mL and 5.8 μg/mL, respectively [130], compared to other *C*. *albicans* and *B*. *cereus* studies.

Solvent selection can influence extract yield and antimicrobial activity, which has been demonstrated by several studies [130,136,140]. For example, the leaves of *E*. *coccineum* extracted by hexane, dichloromethane, ethyl acetate, and ethanol have different MIC values against *E*. *coli* at 250, 31.125, 125, and 250 µg/mL, respectively [136]. However, *M*. *integriflora* leaves have different MIC values by using the same methanol extract: 2790 μg/mL in Mt Coo-tha from the Botanical Gardens (Brisbane, Australia) [141] and 2.4 μg/mL in the Logan campus of Griffith University (Brisbane, Australia) [130], due to the different location of the collection. Thus, the environmental conditions of plant growth also have an impact on the bioactive properties of the plants.

Numerous in vitro studies have been conducted on the antimicrobial activity of Proteaceae species (Table 4).

**Table 4:** Summary of antimicrobial activity of the family Proteaceae.

Species	Bacterium Type	MIC	References
*H*. *salicifolia* (leaf)	*Staphylococcus aureus*, *S*. *epidermidis*, *Enterococcus faecalis Mycobacterium smegmatis*, *Candida albicans*	15–250 μg/mL	[140]
*H*. *salicifolia* (bark)		7.5–250 μg/mL	
*H*. *salicifolia* (fruit)		15–250 μg/mL	
*H*. *sericeae* (leaf)		62–250 μg/mL	
*Banksia genus* (leaf)	*Phytophthora cinnamom*	1–6 mg/mL	[142]
*Roupala brasiliensis* (stem)	*C*. *albicans*, *C*. *glabrata*, *C*. *krusei*, *C*. *parapsilosis*, *C*. *tropicalis*, *Cryptococcus neoformans*, *Escherichia coli*, *E*. *faecalis*, *Klebsiella pneumoniae*, *Pseudomonas aeruginosa*, *S*. *aureus*	15.6–>1000 μg/mL	[138]
*D*. *thouarsii* (leaf)	*Bacillus cereus*, *B*. *megaterium*, *S*. *aureus*, *E*. *faecalis*, *Vibrio harveyi*, *V*. *fisheri*, *Salmonella enterica*, *S*. *antarctica*, *E*. *coli*, *K*. *pneumoniae*	12.5–>100 mg/mL	[135]
*Hakea sericea* (fruit)	*S*. *aureus*, Methicillin-resistant *S*. *aureus*	0.31 mg/mL	[131]
*M*. *integriflora* (flower)	*Aeromonas hydrophilia*, *Citrobacter freundi*, *E*. *coli*, *Proteus mirabilis*, *Pseudomonas flurosecens*, *Serratia marcenscens*, *C*. *albicans*, *Saccharomyces cerevisiae*	2.9–19.9 µg/mL	[130]
*M*. *integriflora (leaf)*	*A*. *hydrophilia*, *C*. *freundi*, *E*. *coli*, *P*. *mirabilis*, *S*. *marcenscens*, *C*. *albicans*, *S*. *cerevisiae*, *B*. *cereus*.	2.4–22.1 µg/mL	
*M*. *integrifolia* (nut)	*P*. *mirabilis*	15 µg/mL	[141]
*M*. *integrifolia* (leaf)		2790 µg/mL	
*E*. *coccineum* (leaf)	*E*. *coli*, *K*. *pneumoniae*, *Proteus mirabilis*, *P*. *aeruginosa*, *S*. *aureus*, *Streptococcus pyogenes*	31.125–500 μg/mL	[136]
*E*. *coccineum* (bark, leaf)	*P*. *aeruginosa*, *E*. *coli*	No inhibition	[139]
*G*. *avellana* (aerial parts)		64–>512 μg/mL	
*Banksia integrifolia* (bark)	*P*. *aeruginosa*, *E*. *coli*, *B*. *cereus*, *S*. *aureus*, *Streptococcus pneumoniae*, *C*. *albicans*	78–1250 µg/mL	[143]
*Bleasdalia bleasdalei* (bark)		78–624 µg/mL	
*Buckinghamia celsissima* (bark)		312–624 µg/mL	
*Cardwellia sublimis* (bark)		<19.5–>2500 µg/mL	
*Darlingia darlingiana* (bark)		39–312 µg/mL	
*D*. *thouarsii* (bark)	*S*. *pyogenes*, *S*. *aureus*, *Clostridium perfringens*, *Listeria monocytogenes*, *P*. *mirabilis*	0.197–0.31 mg/mL	[144]
*Knightia excelsa* (honey)	*E*. *coli*	22.0 ± 4.1 mg/mL	[145]
*Lomatia hirsute* (leaf)	*C*. *albicans*	8 μg/mL	[146]
*O*. *grandiflora* (aerial parts)	*S*. *aureus*	2 mg/mL	[147]
*Roupala* sp. (stem)	*S*. *aureus*, *E*. *faecalis*	60–100 μg/mL	[148]
*B*. *celsissima* (leaf)	*A*. *hydrophilia*, *B*. *cereus*, *B*. *subtilis*, *Citrobacter freundii*, *E*. *coli*, *C*. *albicans*, *S*. *cerevisiae*	8.3–13.6 mm inhibition zone at 0.02 mg/mL	[149]
*Protea rotundifolia* (herb)	*S*. *aureus*, *Micrococcus luteus*	20.5–30.0 μM	[150]
*Toronia toru* (leaf and stem)	*B*. *subtilis*, *E*. *coli*, *P*. *aeruginosa*, *T*. *mentagrophytes*	Inhibition zone of 4-hydroxyphenyl 6-*O*-[(3R)-3,4-dihydroxy-2-methylenebutanoyl]-*β* -d-glucopyranoside: 3–5 mm	[151]
*P*. *linearis* (fruit)	*B*. *subtilis*, *P*. *cinnamomic*, *E*. *coli*	4-hydroxyphenyl 6-*O*-[(3R)-3,4-dihydroxy-2-methylenebutanoyl]-*β*-d-glucopyranoside: 6.25–12.5 μg/disk	[25]
*M*. *integrifolia* (kernel)	*Alternariu heliarzthi*, *Botrytis cinerea*, *Ceratocystis purodoxa*, *Colletotrichum falcutum*, *Fusurium oxysporum*, *Leptosphaeria maculans*, *Macrophomina phaseolinu*, *Phytophthoru cryptogeu*, *Pyrhium grunzinicolu*, *Sclerotinia sclerotiorum*, *Sclerotinia sclerotiorum*, *Sclerotium rolfsii*, *Verticilium dahlia*, *Clavibacter michigunensis*, *P*. *yeudomonus rubrilineans*, *Aspergillus fumigatus*, *Candidu nlhicuns*, *Microsyorum gypseum*, *E*. *coli*, *Saccharonzyces cerevisiae*, *Colletotrichum gloeosporioides*.	MiAMPl peptide: 2–>100 μg/mL	[152]
		MiAMP2c peptide: 5–>50 μg/mL	[153]
*G*. *pteridifolia* (stem)	*B*. *anthraci*, *S*. *simulans*, *Enterococcus faecali*, *Enterococcus faecium*, *L*. *monocytogenes*, *Shigella dysenteriae*, *S*. *epidermidis*, *S*. *aureus*, *S*. *pneumoniae*.	<0.0325–4.0 µg/mL in Kakadumycin A <0.0325–8.0 µg/mL in Echinomycin 0.125–4.0 µg/mL in Vancomycin	[154]
*D*. *thouarsii* (leaf)	*P*. *aeruginosa; V*. *harveyi; V*. *fischeri; Salmonella enterica; S*. *antarctica; E*. *coli; K*. *pneumoniae; B*. *cereus; B*. *megaterium; E*. *faecalis; S*. *aureus*.	7–19 mm inhibition zone at 1 mg/disc	[155]
*F*. *saligna*	*Propionibacterium acnes*	500 µg/mL	[109]
*G*. *juncifolia* (leaf)	*Alcaligenes faecalis*, *Pseudomonas fluorescens*, *Yersinia entercolitica*, *B*. *cereus*, *B*. *subtilis*, *S*. *aureus*, *S*. *epiedermidis*, *Artemia nauplii*	62–1387 µg/mL	[156]
*G*. *juncifolia* (flower)	*A*. *hydrophilia*, *P*. *fluorescens*, *Y*. *entercolitica*, *B*. *cereus*, *B*. *subtilis*, *S*. *aureus*, *S*. *epiedermidis*, *A*. *nauplii*	226–1055 µg/mL	
*G*. *robusta* (leaf)	*A*. *hydrophilia*, *A*. *faecalis*, *P*. *fluorescens*, *Y*. *entercolitica*, *B*. *cereus*, *B*. *subtilis*, *S*. *aureus*, *S*. *epiedermidis*, *A*. *nauplii*, *S*. *Salford*, *K*. *pneumoniae*	83–1788 µg/mL	
*G*. *robusta* (flower)	*B*. *cereus*, *A*. *nauplii*	880–2360 µg/mL	
*G*. *banksia* (inflorescence)	*E*. *coli*	5.0 ± 0.1% inhibition at 250 µg/mL	[157]
*Hakea* spp. (leaf)	*L*. *monocytogenes*, *M*. *luteus*, *S*. *aureus*, *E*. *coli*, *K*. *pneumoniae*, *P*. *aeruginosa*	Neutral to very inhibitory	[129]
*G*. *avellana* (aerial parts)	MRSA, Methicillin-Sensitive *S*. *aureus*	>512 µg/mL	[158]
*E*. *coccineum* (cortex and folium)		>512 µg/mL	
*F*. *saligna* (leaf)	*M*. *tuberculosis*	>1000 µg/mL	[159]
*H*. *sericea* (stem)	*S*. *aureus*, *B*. *cereus*, *L*. *monocytogenes*, *E*. *coli*, *P*. *aeruginosa*, *K*. *pneumoniae*, *methicillin-resistant S*. *aureus*, *C*. *albicans*, *C*. *tropicalis*	0.315–2.5 mg/mL	[160]
*H*. *sericea* (leaf)		0.315–2.5 mg/mL	
*H*. *sericea* (fruit)		0.04–2.5 mg/mL	
*B*. *menziesii* (floral)	*S*. *aureus*, *E*. *faecalis*, *E*. *coli*, *P*. *aeruginosa*	26.8% *w*/*v*	[104]
*B*. *sessilis* (floral)		23.4% *w*/*v*	
*A*. *sericeus* (stem)	*B*. *subtilis*, *S*. *aureus*, *E*. *coli*, *Salmonella* sp.	10–16 mm inhibition zone at 100 mg/mL	[107]
*Alloxylon flammeum* (bark)	*B*. *cereus*, *S*. *aureus*, *P*. *aeruginosa*, *E*. *coli*, *C*. *albicans*, *A*. *niger*	78–1250 µg/mL	[137]
*Athertonia diversifolia* (bark)		312–1250 µg/mL	
*Austromuelleria trinervia* (bark)		156–1250 µg/mL	
*Carnrvonia araliifolia* (bark)		<19.5–1250 µg/mL	
*Darlingia ferruginea* (bark)		156–1250 µg/mL	
*G*. *baileyanna* (bark)		78–1250 µg/mL	
*G*. *hilliang* (bark)		<19.5–625 µg/mL	
*Helicia australasica* (bark)		312–1250 µg/mL	
*Lomatia fraxinifolia* (bark)		39–625 µg/mL	
*M*. *grandis* (bark)		156–625 µg/mL	
*Opisthiolepis heterophylla* (bark)		78–1250 µg/mL	
*Placospermum coriaceum* (bark)		156–1250 µg/mL	
*Stenocarpus sinuatus* (bark)		78–1250 µg/mL	
*Triunia erythrocarpa* (bark)		39–1250 µg/mL	
*E*. *coccineum* (leaf and bark)	*E*. *coli*, *P*. *aeruginosa*	No activity	[139]
*G*. *avellana* (leaf, stem, and fruit)		>512 μg/mL	
*L*. *hirsuta* (leaf and stem)		No activity	
*Protea caffra*	*E*. *coli*, *E*. *faecalis*, *K*. *pneumoniae*, *S*. *aureus*, *Penicillin-resistant S*. *aureus*.	0.31–>2.5 mg/mL	[161]

**Figure 3:**
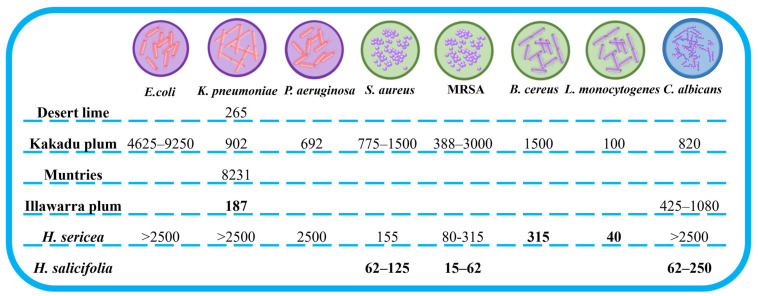
Comparison of antimicrobial activity (MIC; µg/mL) between Proteaceae species and Australian native fruits [124,140,160,166,167,168].

Three Proteaceae species have investigated the antimicrobial activity of their fruits including Gram-positive bacteria, Gram-negative bacteria, and yeast, whilst some Australian native fruits also studied the antimicrobial activity summarized in Figure 3. The fruit of *H*. *sericea* exhibited higher antibacterial activity against Gram-positive bacteria (*Staphylococcus aureus*, MRSA, *Bacillus cereus*, and *Listeria monocytogenes*) than that of Kakadu plum. However, the antibacterial activity against Gram-negative bacteria (*K*. *pneumoniae*, *P*. *aeruginosa*, and *E*. *coli*) in the fruit of *H*. *sericea* is weaker than that of Kakadu plum. The fruit of *H*. *salicifolia* has stronger antimicrobial activity against *Staphylococcus aureus* and MRSA compared with the fruit of Kakadu plum and *H*. *sericea*. *K*. *pneumoniae* as one of the important nosocomial pathogens in paediatric wards is increasing in the number of outbreaks due to drug resistance [162,163]. *Podocarpus elatus* (Illawarra plum) has the strongest activity at an IC_50_ value of 187 µg/mL against *K*. *pneumoniae*, followed by Desert lime (265 µg/mL), Kakadu plum (902 µg/mL), *H*. *sericea* (2500 µg/mL), and Muntries (8231 µg/mL), respectively (Figure 3). Thus, Australian native fruits have promising potential applications in the treatment of *K*. *pneumoniae* infections. Furthermore, *Candida* spp. is one of the major and few fungal species that cause human diseases living in the healthy human body without pathogenicity, but it could cause serious infections in immunocompromised individuals [164]. *Candida* spp. is still a serious medical problem leading to a high death rate and frequently a nosocomial infection [165]. One of the reasons is drug resistance. *H*. *salicifolia* fruit has higher antifungal activity against *C*. *albicans* compared to Kakadu plum, riberry, muntries, and *H*. *sericea* (Figure 3). Thus, it is a good opportunity to develop plant-based antifungal agents using Australian native species. However, there is still a lack of information on the antimicrobial activity of Australian native fruits, which need to be further investigated to potentially develop new antimicrobial agents.

### 6.3. Cytotoxicity

Cancer is one of the major health problems worldwide. According to the World Health Organization (WHO), an increase in the number of deaths caused by cancer was presented from about 4 million in 2014 to 10 million in 2018 [169]. Also, in excess of 60% of natural products (more than 3000 plants) were considered as being the source of value-added anticancer medicines [170]. Thus, interest in the discovery of anticancer agents has increased and developed further. Cytotoxicity research is an in vitro study to screen the cell growth/damage and its reproduction treated by medical agents. There are four main cytotoxicity assays commonly used today: dye exclusion, colorimetric assays, fluorometric assays, and luminometric assays [171]. The methyl thiazolyl tetrazolium (MTT) assay, as one of the colorimetric cell proliferation assays, is the most performed in studies. However, the MTT assay is too sensitive, leading to application problems. For example, (-)-epigallocatechin-3-gallate and kaempferol can reduce the MTT to formazan interfering with the results [172].

Different types of assays were used in the cytotoxic analysis of the Proteaceae species, including trypan blue dye [173], the MTT assay [98,151,155,174,175,176,177,178,179], XTT assay [159], MTS assay [143,180,181,182], WST-1 assay [38], brine shrimp lethality assay [138,183,184], resazurin reduction assay [185], and fluorescein diacetate assay [152]. Most of the studies chose HepG2, the isolation of hepatocellular carcinoma, to be considered as the cell line. This might be because liver cancer (20.3%) is one of the urgent health issues, third only to the pancreas (11.5%) and esophagus (20.6%) cancers in survival rate globally [186]. The bark of *Buckinghamia celsissima* has the notably highest anti-proliferative effect at an IC_50_ value of 4.43 µg/mL [143] compared to other Proteaceae species against the HepG2 cell line as published before. There are only four studies investigating breast tumours (MCF-7 and MDA-MB-231) in *G*. *robusta*, *B*. *bleasdalei*, *Cardwellia sublimis*, *C*. *araliifolia*, *M*. *grandis*, and *O*. *heterophylla*, although breast cancer has the highest incidence rate worldwide compared to other cancers [169]. According to the current published studies of the Proteaceae family, the inner stem of *K*. *excelsa* exhibited excellent anticancer activity against P388 at an IC_50_ value lower than 1 µg/mL [187]. Moreover, the leaves and barks were chosen as study materials in most anticancer studies of the Proteaceae family rather than the fruits, contributing to a gap in cytotoxic studies.

Overall, around half of the cytotoxicity studies in the family Proteaceae investigated the activity of their compounds, of which some compounds have been investigated previously in other families. 2-methoxyjuglone has been found in approximately 20 species of the *Juglandaceae*, *Sterculiaceae*, and Proteaceae families as summarized previously [188]. This compound showed a cytotoxicity against HepG2 cells with an IC_50_ value of 3.8 µg/mL in leaves of *L*. *hirsute* [174], which was weaker than the study with an IC_50_ value of 2.2 µg/mL [189]. Besides that, 2-methoxyjuglone showed in vitro antitumor activity against a range of human cancer cells, mainly studied in breast cancer cells, colon adenocarcinoma cells, and hepatocellular carcinoma cells [188]. Graviquinone is another cytotoxic compound found in *G*. *robusta* against MCF-7 (IC_50_: 15.0 ± 3.0 μM), NCI-H460 (10.8 ± 2.3 μM), and SF-268 cell lines (5.9 ± 0.1 μM) [189]. It also has cytotoxic abilities against other cell lines, including thymic lymphoma, lung tumour, immortalized cells, and squamous cell carcinoma ranging from IC_50_ values of 0.03 to 11.83 μM [190]. Thus, graviquinone could be a remarkable cytotoxic compound in the treatment of many tumours. Methyl 2,5-dihydroxycinnamate, as one of the compounds in *G*. *robusta*, has previously been reported in the leaves and branches of *Philadelphus coronaries* [191] and the leaves of *Murraya paniculate* [192] with potential cytotoxic effects against a range of tumours. Hydroquinone, from the leaves of *H*. *lobata*, inhibited the MGC-803 and HEEC cell lines at 11.3 ± 2.1 and 19.4 ± 1.9 µg/mL, respectively [176]. This compound has been reviewed and found in land and marine plants summarized in studies of cytotoxicity and antioxidant activity [193,194,195].

Table 5 summarized the cytotoxicity of the family Proteaceae and only 24 species have been studied, which makes up approximately 1% of the whole Proteaceae species.

**Table 5:** Summary of cytotoxicity of the family Proteaceae.

Species: Compounds/Extract		Cell Line	IC_50_	References
*B*. *bleasdalei* (bark): (24*E*)-3*β*-hydroxy-7,24-euphadien-26-oic acid		P388	About 80% of viable cells at 25 µM	[173]
*L*. *hirsute* (leaf): 2-methoxyjuglone		HepG2	3.8 µg/mL	[174]
*F*. *saligna* (leaf)		U937	202.4 µg/mL	[159]
*Kermadecia elliptica* (bark): Kermadecin A–D		L1210	4.1–18.5 µM	[175]
		KB	3.6–>10 µM	
*T*. *toru* (leaf): 4-hydroxyphenyl 6-*O*-(4-hydroxy-2-methylenebutanoyl)-*β*-d-glucopyranoside, 4-hydroxyphenyl 6-*O*-[(3R)-3,4-dihydroxy-2-methylenebutanoyl]-*β*-d-glucopyranoside, arbutin		P388	50–100 µg/mL	[151]
		BSC	3–>25 µg/mL	
*G*. *robusta* (leaf): Graviquinone, cis-3-hydroxy-5-pentadecylcyclohexanone, methyl 5-ethoxy-2-hydroxycinnamate, methyl 2,5-dihydroxycinnamate		MCF-7	15.0–>50 µM	[180]
		NCI-H460	10.8–>50 µM	
		SF-268	5.9–>50 µM	
*B*. *integrifolia* (bark)		HepG2	20.66 µg/mL	[143]
*B*. *bleasdalei* (bark)		HepG2	46.20 µg/mL	
		MDA-MB-231	61.23 µg/mL	
*B*. *celsissima* (bark)		HepG2	4.43 µg/mL	
*Cardwellia sublimis* (bark)		HepG2	94.62 µg/mL	
		MDA-MB-231	100 µg/mL	
		Human 5637	32.57 µg/mL	
*D*. *darlingiana* (bark)		HepG2	42.20 µg/mL	
		5637	12.40 µg/mL	
*H*. *lobata* (leaf): 6′-((E)2-methoxy-5-hydroxycinnamoyl) arbutin, 2′-((E)2, 5-dihydroxycinnamoyl) arbutin, 6′-[(E)-2″-hydroxymethyl-2″-butenylacyl] arbutin, 6′-[(E)-4″-hydroxycinnamoyl] arbutin, 6′-[(E)-2″, 5″-dihydroxycinnamoyl] arbutin, grevillic acid, hydroquinone.		MGC-803	11.3 ± 2.1–>50 µg/mL	[176]
		HEEC	19.4 ± 1.9–>50 µg/mL	
*K*. *elliptica* (bark): kermadecin A, 17-methoxykermadecin A, 22-methoxykermadecin A, 17,22-methoxykermadecin A, 17,19,22-trimethoxykermadecin A, (±)-cis-27,28-dihydroxy-17,19,22-trimethoxy-27,28-dihydrokermadecin A, (±)-28-hydroxy-17,19,22-trimethoxy-27-oxo-27,28-dihydrokermadecin A, (±)-cis-27,28-diacetoxy-17,19,22-trimethoxy-27,28-dihydrokermadecin A, 27,28-dihydrokermadecin A, 17,19,22-trimethoxy-27,28-dihydrokermadecin A		U937	3.86–>100 µM	[181]
		HL60	2.29–100 µM	
		KB	2.78–100 µM	
*D*. *thouarsii* (leaf): Dilobenol A–G		FcB1	15.8 ± 1.4–34.3 ± 0.6 µM	[155]
		L-6	58.8 ± 0.4–>137 µM	
*G*. *robusta* (leaf): Gravicycle, Dehydrogravicycle, Bisgravillol, Dehydrobisgravillol, Dehydrograviphane, Methyldehydrograviphane, Graviphane, Methylgraviphane, Robustol, dehydrorobustol A, bis-norstriatol, 5-[14′-(3″,5″-dihydroxyphenyl)-*cis*-tetradec-6′-en-1-yl] resorcinol, *cis*-5-n-pentadecylresorcinol, *cis*-5-n-pentadec-8′-enylresorcinol		MCF-7	28.6 ± 3.2–37.1 ± 1.9 µM	[182]
		NCI-H460	22.8 ± 1.3–35.4 ± 1.7 µM	
		SF-268	27.7 ± 1.5–39.2 ± 0.7 µM	
*O*. *grandiflora* (leaf)		HL-60	3.12–6.25 µg/mL	[38]
*O*. *grandiflora* (flower)			50–100 µg/mL	
*Protea madiensis* (root and bark)		LOCE-MM001	10.0–>500 µg/mL	[177]
		LOCE-MM028	10.0–>500 µg/mL	
*R*. *brasiliensis* (stem)		BS	197.7–>1000 µg/mL	[138]
*G*. *robusta* (leaf)		BS	0.45 ± 0.04–191.14 ± 0.19 µg/mL	[183]
*K*. *excelsa* (inner stem)		P388	<1 µg/mL	[187]
*H*. *erratica* (seed)		BS	>1000 µg/mL	[184]
*G*. *robusta* (aerial part)		WI-38	249.5 ± 10.7 µg/mL	[178]
		MCF-7	89.5 ± 6.3 µg/mL	
		HepG2	199.1 ± 25.7 µg/mL	
*G*. *whiteana*: NP-011694, NP-013296, NP-013330, NP-013378, NP-014428		L6	15.5 ± 1.8–54.2 ± 0.5 µM	[196]
*H*. *salicifolia* (leaf)		RAW 264.7	>900 µg/mL	[185]
*Telopea speciossissima* (leaf)		RAW 264.7	>900 µg/mL	
*C*. *teretifolium/C*. *brownie* (root): 3-geranyllawsone		U373	48 μM	[179]
		Hs683	12 μM	
		A549	11 μM	
		PC-3	28 μM	
		SKMEL-28	12 μM	
		LoVo	7 μM	
*H*. *terminalis* (trunk)		HepG2	99.6 ± 5.0% inhibition (b)	[98]
*M*. *integrifolia* (nut): MiAMPl peptide		Hela	>1 mg/mL	[152]
Bark	*A*. *flammeum*	Hs578T	100% (a)	[137]
	*A*. *diversifolia*	SK-MEL-28	38.56% (a)	
	*A*. *trinervia*	SK-MEL-28	58.58% (a)	
	*C*. *araliifolia*	MDA-MB-231, 5637	100% (b)	
	*D*. *ferruginea*	SK-MEL-28	19.86% (a)	
	*G*. *baileyanna*	5637	40.20% (b)	
	*G*. *hilliang*	Hs578T	99.41% (a)	
	*H*. *australasica*	HepG2	55.32% (a)	
	*L*. *fraxinifolia*	Hs578T	99.47% (a)	
	*M*. *grandis*	MDA-MB-231	44.50% (b)	
	*O*. *heterophylla*	MDA-MB-231	45.03% (b)	
	*P*. *coriaceum*	HepG2	35.61% (a)	
	*S*. *sinuatus*	5637	99.94% (b)	
	*T*. *erythrocarpa*	HepG2	90.11% (a)	

U937: human histiocytic lymphoma; L1210: mouse lymphocytic leukemia; KB: human epithelial carcinoma; P-388: lymphoma; BSC: monkey kidney; NCI-H460: large-cell cancer of the lung; MCF-7: human breast adenocarcinoma; NCI-H460: non-small-cell lung cancer; SF-268: glioblastoma; HepG2: hepatocellular carcinoma; MDA-MB-231: breast adenocarcinoma; 5637: human primary bladder carcinoma; MGC-803: human gastric carcinoma; HEEC: human endometrial epithelial; human histiocytic lymphoma; HL60: human promyelocytic leukemia; FcB1: chloroquine-resistant strain FcB1 of Plasmodium falciparum; L-6: rat myoblast; LOCE-MM001: melanoma human cell; LOCE-MM028: normal human melanocyte; BS: Brine shrimp; MRC-5: human lung; P388: murine leukemia; WI-38: human lung fibroblast cell-line; RAW 264.7: monocyte/macrophage-like cells; U373: human glioblastoma; Hs683: human brain tumour; A549: lung cancer; SKMEL-28: melanoma; LoVo: colon cancer; PC-3: prostate cancer; Hela: immortal cancer; Hs578T: breast ductal carcinoma; SK-MEL-28: malignant melanoma; (a): 250 µg/mL of extract; (b): 100 µg/mL of extract.

### 6.4. Anti-Inflammatory Activity

Inflammation is a biological process to defend against harmful stimuli leading to some regular events, such as redness, diarrhea, swelling, pain, or even loss of function [197]. There are two types of inflammation: acute and chronic inflammation. Although inflammation is the way to defend foreign organisms in the human body, more than 99% of inflammation disorders are severe and serious or even contribute to death, for example, asthma [197]. Currently, synthetic molecular drugs (steroidal and nonsteroidal) are used in the treatment to reduce pain and inflammation but with regular toxic and adverse effects [197,198]. For example, aspirin and mefenamic acid could lead to gastric effects, like bleeding, diarrhea, and gastric erosion. Thus, plant-based anti-inflammatory agents with fewer side effects have been explored in many modern pharmaceuticals, which are also popularly used by Aboriginal peoples. For instance, *Eucalyptus* genus is one of the plants used as anti-inflammation agents by the Dharawal Aboriginal people [199]. There are ten inhibition mechanisms of cellular action applied in anti-inflammation activity: hypothalamic–pituitary–adrenal (HPA)-dependent anti-inflammatory drugs, arachidonic acid related enzymes, cytokines, signalling pathways, vasoactive mediators, nitric oxide (NO), reactive oxygen species (ROS), inflammatory mediators, immunological regulation, and gut microbiota [200,201].

The inhibitions of NO, COX-1/2, XO, LOX, and tumour necrosis factor-α (TNF-α) are several mechanisms of action used in the analysis of the Proteaceae family (Table 6). Animals as the study target were also applied to evaluate anti-inflammatory activity, which was usually applied to the target cells directly. In NO expression, the roots of *H*. *terminalis* exhibited the highest anti-inflammatory activity with an IC_50_ value of 11.98 ± 0.71 µg/mL among the published Proteaceae species (*F*. *saligna*, *H*. *terminalis*, *H*. *salicifolia*, and *T*. *speciossissima*). In animal model studies, different animals were used in the anti-inflammatory activity of some Proteaceae species (*F*. *speciosa*: chicks; *L*. *hirsute* and *G*. *robusta*: pigs; *G*. *robusta* and *L*. *hieronymi*: rats). These animal models are commonly used in anti-inflammatory studies by inhibiting animal edema [201]. However, there is no comparability of anti-inflammatory activity among different Proteaceae species due to the different study models. Also, multiple study models can contribute to a more persuasive result.

Different extraction methods, sample processing, solvent use, and sample location have an influence on their bioactivity, which was also illustrated in the anti-inflammatory activity. *P*. *simplex* extracted by different solvents and showing a range of inhibition at the same concentration [202]. Additionally, different sample roasting processes in a *G*. *avellana* study led to a variation in inhibition activity [99]. Thus, it is important to investigate the effects of sample processing to provide a better understanding for further studies.

Only two studies of *H*. *terminalis* [98] *and L*. *hieronymi* [203], focused on the anti-inflammation of phytochemicals. Bisresorcinol found in the trunk of *H*. *terminalis* has anti-inflammatory activity in RAW 264.7 cells at an IC_50_ value of 71.15 ± 6.66 mg/mL and anti-aging activity against collagenase, elastase, and tyrosinase at 156.7 ± 0.7, 33.2 ± 0.5, and 22.8 µM/L, respectively [98]. The attractive forces of π-electrons and hydrogen bonds between the bisresorcinol and enzyme could be the main responsibility for the anti-aging activity. Bisresorcinol and its derivatives are also found in the stems of *Grevillea glauca* [204]. Oleanolic acid, another compound found in the Proteaceae species, has been investigated and reviewed before with plenty of studies and patents to be considered as a potent anti-inflammatory agent from 1980 to the present [205,206]. It is recommended that a further in-depth investigation should be focused on the compounds of the plants to discover their potential anti-inflammatory agents.

All anti-inflammatory activity studies of the Proteaceae family have selected the leaves, flowers, stems, roots, nuts, honey, barks, and trunks to be the study materials, excluding the fruits. Thus, further studies are necessary to include diverse study materials to obtain comprehensive results to fill the knowledge gap.

**Table 6:** Summary of anti-inflammatory activity of the family Proteaceae.

Species	Study Mode	IC_50_	References
*O*. *grandiflora* (leaf)	ROS	4.1 ± 0.07 µg/mL	[38]
*O*. *grandiflora* (flower)		5.87 ± 1.48 µg/mL	
*F*. *speciosa* (leaf)	Animal	55.50 ± 0.78% inhibition at 100 mg/kg	[102]
*F*. *saligna* (bark)	NO	21.0 ± 0.7 µg/mL	[110]
*H*. *terminalis* (trunk): Bisresorcinol	NO	71.15 ± 6.66 mg/mL	[98]
*P*. *simplex* (bark)	COX-1	86.1–94.2% inhibition at 250 μg/mL	[202]
	COX-2	16.7–41.0% inhibition at 250 μg/mL	
*P*. *simplex* (leaf)	COX-1	57.8–100.1% inhibition at 250 μg/mL	
	COX-2	20.9–72.4% inhibition at 250 μg/mL	
*L*. *hirsuta* (leaf)	Animal	17.1 ± 0.8% inhibition at 4.0 mg/kg	[58]
*G*. *robusta* (leaf)	Animal	6.2 mm thickness at 400 mg/kg	[207]
*G*. *robusta* (bark)		6.8 mm thickness at 400 mg/kg	
*L*. *hieronymi* (stem): oleanolic acid, epi-oleanolic acid, epi-Maslinic acid, p-Hydroxyacetophenone, p-Hydroxyacetophenone-β-glucoside	Animal	24–30% inhibition at 80 mg/kg	[203]
*P*. *falcata* (leaf)	XO	2.24 ± 1.72% inhibition at 100 µg/mL	[208]
*K*. *excelsa* (honey)	15-LOX	>2000 µg/mL	[209]
*H*. *salicifolia* (leaf)	NO	195.9 ± 30.7 µg/mL	[185]
	TNF-α	697.7 ± 185.3 µg/mL	
*T*. *speciossissima* (leaf)	NO	116.5 ± 20.1 µg/mL	
	TNF-α	555.1 ± 87.5 µg/mL	
*H*. *terminalis* (root)	NO	11.98 ± 0.71 µg/mL	[210]
*G*. *avellana* (nut)	COX-1	50.1–79.9% inhibition at 100 µg/mL	[99]
	COX-2	15.7–33.8% inhibition at 100 µg/mL	
	LOX	9.1–26.0% inhibition at 100 µg/mL	

NO: neutrophil oxidation; LOX: lipoxygenase; 15-LOX: 15-lipoxygenase; COX-1&2: cyclooxygenases-1&2; XO: xanthine oxidase; ROS: reactive oxygen species.

### 6.5. Antiviral Activity

Due to the genetic variation of viruses, a pandemic event usually occurs in a human lifetime. Although people have developed several antiviral medicines in the past, many drugs cannot effectively target viruses due to the poor properties of some conventional drugs and viral resistance. Thus, there is an increased interest in the use of plant materials for the treatment of viral infections. Around 100 species have been reviewed in previous studies primarily focusing on the herpes simplex virus (HSV), human immunodeficiency virus (HIV), influenza virus, and hepatitis C virus (HCV) [211].

Some Proteaceae species have been studied for their antiviral activity. *Conospermun incurvum* can inhibit the influences of HIV-1_RF_ to protect the T4-lymphoblastoid cell line, especially a naphthoquinone derivative, conocurvone, found in *C*. *incurvum* [212]. The barks of *D*. *darlingiana* and *B*. *bleasdalei* showed potential activity against HSV [143] and plants without the roots of *Hakea saligna* against Ranikhet disease virus at the LD_50_ value of >1000 mg/kg [213]. There is no antiviral activity found in the leaves of *Lomatia ferruginea* against HSV and HIV, and the barks of *Banksia integrifolia*, *Cardwellia sublimis*, and *Buckinghamia celsissima* against HSV. Currently, only HIV, HSV, and the Ranikhet disease virus are known to be inhibited by some Proteaceae species, though studies are limited. Compared with other in vitro bioactive studies, antiviral studies are very limited in the family Proteaceae.

### 6.6. Other Bioactivities

There are other bioactive properties studied in the family Proteaceae, which also could contribute to human health and benefits. Food safety is important for human health, and many diseases originate from food sources due to the presence of parasites. Many parasitic diseases are from the tropics, such as malaria, trypanosomiasis, leishmaniasis and schistosomiasis, which can cause serious diseases and health disorders. Numerous plant families have been used for parasitic treatment, such as Moraceae, Myrtaceae, Papaveraceae, and Rutaceae [214]. The Proteaceae family is one of the potential plants in parasitic treatment for human beings. Currently, only *Schistosoma mansoni*, *Leishmania* spp., and malaria have been selected in the antiparasitic study of the family Proteaceae. The aerial parts of *R*. *montana* showed antiparasitic activity against *S*. *mansoni* as well as botulin, quercetin-3-*O*-β-d-glucoside, and quercetin-3-*O*-β-d-rhamnoside have been isolated from the plant [215]. The timber of *G*. *robusta* and *O*. *grandiflora* displayed in vitro antiparasitic activity against *Leishmania promastigotes* at MIC values of 50 and 23.7 µg/mL, respectively [38,216]. Malaria can cause extremely dangerous diseases and is one of the major health problems worldwide. Two species from the family Proteaceae have been studied in antimalarial studies. The leaves and twigs of *Faurea speciosa* exhibited promising inhibitory activity against *Plasmodium* spp. [102,217,218]. Also, *L*. *concinnum* has been traditionally used in the treatment of malaria [219]. It has been indicated that the Proteaceae species have the potential of application in antiparasitic activity to improve drug resistance and develop novel plant-based products. Moreover, two studies focused on melanogenesis inhibitory activity using *G*. *robusta* and *Serruria furcellata*, which could be due to the high value of arbutin derivatives [220,221]. Thus, these two species have the potential of being applied in skin-lightening and anti-chloasma agents. It has also been proved that the arbutin and its derivatives could be found in more potential native species and to be used as skin-lightening agents. *S*. *furcellata* from the family Proteaceae showed tyrosinase inhibitory activities to inhibit the biosynthesis of melanin [221] and could be used as a potential inhibitor of freckles. *Kermadecia rotundifolia* was active against acetylcholinesterase [222] and could be used as an acetylcholinesterase inhibitor to replace Western medicines and reduce side effects from chemical medicines. The Proteaceae species also have the ability to treat cardiovascular diseases. One study described striatal isolated from *G*. *robusta* and *G*. *striata* as a potent inhibitor of plasma membrane Ca^2+^-ATPase, which can be used for stimulating a failing heart [223]. Although many Proteaceae species exhibited plant-based values in various aspects, preliminary studies on antiparasitic, melanogenesis inhibitory, and potential medical applications are limited. Based on promising bioactivities in this study, the discovery of novel plant-based food and pharmaceutical products from the family Proteaceae is essential for human health consumption.

## 7. Bioactive Properties of the *Persoonia* Genus

Table 7 shows the previously published studies of the bioactive properties of *Persoonia* spp., which demonstrate that *Persoonia* fruits have exhibited excellent antimicrobial activity against a wide range of bacteria [25]. Only one study describing the toxicity of *P*. *pinifolia* reported that the fruit extracted by chloroform led to the death of mice, which could be because the compounds were unable to be completely purified [24]. A few studies have reported novel chemical compounds in *Persoonia* spp. including arbutin, arbutin derivatives, and (*Z*)-5-undec-3-enylresorcinol. Arbutin and its derivatives have been proven to have antioxidant, antimicrobial, anti-aging, and anti-inflammatory activities [224,225,226]. For example, arbutin-rich *Arbutus unedo* leaves exhibited antioxidant properties in the treatment of gastrointestinal complaints [227]. Moreover, anthocyanidin has also been found in several *Persoonia* species [228]. It could have potential bioactive properties, such as anti-inflammatory, antioxidant, and antimicrobial activity [229].

## 8. Opportunities and Challenges

There are many potential economic, cultural, and social benefits to the development of Australian indigenous species. From an economic aspect, Australian indigenous species provide huge marketing value. The Aboriginal peoples rely on their plants to meet the needs of their diet and medicines. For example, edible fruit, nut, gum, and honey from numerous Proteaceae species have been eaten by Australian Aboriginal peoples and some species have been commercialized. The *Persoonia* fruit is a favourite snack food during the childhood of the Kambuwal people of southeastern Queensland. Also, as mentioned before, over 50,000 tons of *Macadamia* nuts from Australia were exported in 2020 with an increased export trend in the Australian Macadamia market from 2014 to 2020 [35]. They could contribute to commercial opportunities for Australian native food. From a social aspect, the development of Australian native species provides a connection to the country and traditional food for Aboriginal people, as these species are normally harvested by remote and rural communities. To develop Australian native value chains, further investigation of harvesting, processing, and storage conditions of native fruit is required. Regulatory approval is critical to meeting both domestic and international trade obligations. Although some legislation has been documented about threatened species and the protection of habitats, there are some ineffective regulations in the EPBC Act and the Biodiversity Conservation Act 2016, leading to an increase in the extinction rate of Australian native species. Thus, this area needs to be further assessed to improve the protection of Australian native species and the prevention of biodiversity loss [232,233].

The development of Australian indigenous species can improve food insecurity. Currently, food and nutritional security are challenging issues and play a vital role in continuous socioeconomic development in the world. Since the COVID-19 pandemic occurred, food security has become a more serious issue in the world and native food is a good option to improve food insecurity. For example, edible parts from the Proteaceae species can be applied in value-added food sources or ingredients providing food and economic returns to the relevant communities. Nevertheless, there is still limited information on the nutritional and functional properties of Proteaceae fruit for it to be safe for consumption. So, more in vitro and in vivo studies are necessary to further investigate its nutritional and health values. Moreover, the Australian Food Composition Database (https://www.foodstandards.gov.au (accessed on 4 August 2023)) is a comprehensive reference website to provide information on the nutrient content of Australian food. Although numerous native fruits have been studied and the results published, the food composition of native fruit including Proteaceae fruit has not been included in the Australian Food Composition Database. Moreover, different methods and units have been used in the analysis of the nutrient components and bioactivities of indigenous fruit in the literature, which contributes to the difficulty of comparing different fruits to obtain a better understanding of their nutritional and bioactive properties. Also, a lack of reproducibility has been found in studies on the family Proteaceae as their bioactive properties were determined in different botanic parts rather than the same parts. Different maturity stages and fruit processing methods also influence the nutritional level of indigenous fruits and need to be studied further.

## 9. Conclusions and Recommendations for the Future

The demand for herbal plants worldwide is on the rise due to their mineral side effects, ease of local harvest, and lower prices. Various herbal plants are being used for the production of new medicines, ensuring food security and providing diet diversity. Many species of Proteaceae have potential applications in a wide range of industries. Traditionally, these plants have been used as foods and medicines in Australian Aboriginal communities, but pharmacokinetic studies and clinical studies are required to evaluate their safety, efficacy, and bioavailability. In the Proteaceae family, phytochemical studies are still lacking, and only a few species have been quantified. However, there is limited phytochemical information available for the fruit parts of Proteaceae species. Several nutritional and bioactive studies have been conducted on Proteaceae species, however, sample location, harvest period, extraction method, and solvent choice affect plant bioactivity. Further investigation of these influences is necessary in order to maximize the production of bioactive properties from natural plant sources. Different tissues of plants in the Proteaceae family could be further explored for potential application in functional foods and as indigenous products. *Persoonia* spp. has shown promising antimicrobial and anti-inflammatory properties, which could be exploited in the future as functional ingredients or nutraceuticals. It is recommended to conduct additional nutrition and bioactivity tests on *Persoonia* species. Overall, Proteaceae fruit is promising as a food that can contribute to food consumption, environmental sustainability, and socioeconomic development, but further studies are needed to provide safety information.

## Figures and Tables

**Table 2:** Summary of phytochemicals isolated from the family Proteaceae.

No	Compounds	Molecular Formula	Accurate Mass (*m/z*)	Species	References
1	3,4-bis(4-hydroxybenzoyl)-1, 5-anhydro-d-glucitol	C_20_H_20_O_9_	404.37	*P*. *cynaroides*	[57]
2	4-hydroxybenzoyl-1,5- anhydro-d-glucitol	C_13_H_16_O_7_	284.26		
3	2-(hydroxymethyl)-4- oxo-4*H*-pyran-3-yl- 6-*O*-benzoate-β-d- glucopyranoside	C_19_H_20_O_11_	424.36		
4	3-hydroxy-7,8-dihydro -β-ionone-3-*O*-β -d-glucopyranoside	C_19_H_32_O_8_	388.46		
5	3,4-dihydroxybenzoic acid	C_7_H_6_O_4_	154.12		
6	3-hydroxykojic acid	C_6_H_6_O_5_	158.11		
7	B-type Procyanidin	C_30_H_26_O_12_	578.53		[84]
8	Diosmetin	C_16_H_12_O_6_	300.27		
9	3,5-dihydroxy cinnamate	C_10_H_10_O_4_	194.19	*G*. *robusta*	[85]
10	6-hydroxy coumarin	C_9_H6O_3_	162.14		
11	*p*-hydroxybenzeldahyde	C_7_H6O_2_	112.12		
12	Methyl gallate	C_8_H_8_O_5_	184.15		
13	Arbutin 6″-*O*-3,5- dihydroxycinnamic acid	C_21_H_22_O_10_	434.40		
14	Ethyl gallate	C_9_H_10_O_5_	198.17		
15	Icariside B_1_	C_19_H_30_O_8_	386.44		[78]
16	Heliciopside A	C_26_H_28_O_14_	564.50	*H*. *terminalis*	[79]
17	Heliciopside B	C_26_H_28_O_14_	564.50		
18	Heliciopside C	C_28_H_31_O_16_	624.55		
19	Heliciopside D	C_28_H_36_O_16_	628.58		
20	Heliciopside E	C_42_H_42_O_22_	898.78		
21	Clemochinenoside D	C_27_H_30_O_15_	594.52		
22	3,4,5-trimethoxyphenyl-β -d-glucopyranoside	C_15_H_22_O_9_	346.33		
23	Kusukuenol B_1_	C_30_H_44_O_4_	468.68		
24	Ursolic acid	C_30_H_48_O_3_	456.71		[80]
25	3,7,11,15-Tetramethyl-2-hexadecene	C_20_H_40_	280.54	*Stenocarpus sinuatus*	[81]
26	Neophytadiene	C_20_H_38_	278.52		
27	Phytol	C_20_H_40_O	296.54		
28	*α*-Tocospiro A	C_29_H_50_O_4_	462.71		
29	2-Methyloctacosane	C_29_H_60_	408.8		
30	*β*-tocopherol	C_28_H_48_O_2_	416.69		
31	Hentriacontane	C_31_H_64_	436.85		
32	*γ*-sitosterol	C_29_H_50_O	414.72		
33	*β*-sitosterol	C_29_H_50_O	414.72	*M*. *integrifolia*	[82]
34	Monogalactosyl diacylglycerol	C_38_H_72_O_10_	688.51		
35	Protochatechuic acid	C_7_H_6_O_4_	154.12		
36	Kaur-16-ene	C_20_H_32_	272.48	*R*. *montana*	[86]
37	Kaur-15-ene	C_20_H_32_	272.48		
38	Nerolidol	C_15_H_26_O	222.37		
39	Farnesylacetone	C_18_H_30_O	262.44		

**Table 7:** Summary of bioactive properties of *Persoonia* spp.

Bioactive Properties	Findings	References
Treatment	Sore eyes, sore throats, colds, diarrhea, and chest infections treated by leaves and wood of *P*. *falcata*	[28,49,56]
Antimicrobial activity	Potential antimicrobial activity against a wide range of bacteria found in the leaves of *P*. *gunnii*, the fruit of *P*. *pinifolia*, and *P*. *linearis*	[24,25,26]
	Antibiotic activity of *P*. *juniperina* against typhoid bacilli, staphylococci, and *Mycobacterium phlei*	[27]
Toxicity	Mice died injected with the smallest dose of 500 milligrams per kilogram of body weight of chloroform extract of *P*. *pinifolia*	[24]
Anti-inflammatory activity	*P*. *falcata* leaves can against XO given 2.24 ± 1.72% inhibition at 100 µg/mL	[208]
Phytochemicals	Arbutin derivatives and pyroside found in the leaves of *P*. *gunnii* and Arbutin derivatives found in the ripening fruit of *P*. *linearis* × *pinifolia* and *P*. *salicina*	[25,26,230]
	(Z)-5-undec-3-enylresorcinol compound extracted from the wood of *P*. *elliptica*	[231]
	Anthocyanins found in *P*. *linearis*, *P*. *pinifolia*, and *P*. *myrtilloides*	[228]
	Saponins and tannins screened in *P*. *falcata*	[50]

## Data Availability

Not applicable.

## References

[1] Walsh F., Douglas J. (2011). No bush foods without people: The essential human dimension to the sustainability of trade in native plant products from desert Australia. Rangel. J..

[2] Pearson G.J. (2010). Managing the landscapes of the Australian Northern Territory for sustainability: Visions, issues and strategies for successful planning. Futur. J. Policy Plan. Futures Stud..

[3] Ferguson M., Brown C., Georga C., Miles E., Wilson A., Brimblecombe J. (2017). Traditional food availability and consumption in remote Aboriginal communities in the Northern Territory, Australia. Aust. New Zealand J. Public Health.

[4] AIHW Poor Diet. https://www.aihw.gov.au/reports/food-nutrition/poor-diet/contents/dietary-guidelines.

[5] Prakash V., Martín-Belloso O., Keener L., Astley S., Braun S., McMahon H., Lelieveld H. (2016). Regulating Safety of Traditional and Ethnic Foods.

[6] Richmond R., Bowyer M., Vuong Q. (2019). Australian native fruits: Potential uses as functional food ingredients. J. Funct. Foods.

[7] Kubitzki K. (2014). Flowering Plants: Eudicots.

[8] Christenhusz M.J.M., Byng J.W. (2016). The number of known plants species in the world and its annual increase. Phytotaxa.

[9] Goldingay R.L., Carthew S.M. (1998). Breeding and Mating Systems of Australian *Proteaceae*. Aust. J. Bot..

[10] Jussieu A.L.d. (1789). Genera Plantarum Secundum Ordines Naturales Disposita.

[11] Weston P.H. (1995). Flora of Australia Volume 16, Elaeagnaceae, Proteaceae 1.

[12] Westonand P., Barker N. (2006). A new suprageneric classification of the *Proteaceae*, with an annotated checklist of genera. Telopea.

[13] Offord A., Rollason A., Frith C.A. (2015). Tissue culture of *Persoonia* species for horticulture and restoration. Acta Hortic..

[14] Baines J.A. (1981). Australian Plant Genera: An Etymological Dictionary of Australian Plant Gener.

[15] Weston P.H. (2003). *Proteaceae* Subfamily Persoonioideae. Aust. Plants.

[16] Emery N.J., Offord C.A. (2018). Managing *Persoonia* (*Proteaceae*) species in the landscape through a better understanding of their seed biology and ecology. Cunninghamia.

[17] Bellard C., Leclerc C., Hoffmann B.D., Courchamp F. (2015). Vulnerability to climate change and sea-level rise of the 35th biodiversity hotspot, the Forests of East Australia. Environ. Conserv..

[18] WAH Persoonia Sm. https://florabase.dpaw.wa.gov.au/browse/profile/21322.

[19] Rymer P.D. (2006). Plant Rarity: Species Distributional Patterns, Population Genetics, Pollination Biology, and Seed Dispersal in *Persoonia* (*Proteaceae*). Ph.D. Thesis.

[20] DCCEEW Species Profile and Threats Database: Persoonia. https://www.environment.gov.au/cgi-bin/sprat/public/spratlookupspecies.pl?proc=search&searchtype=wildcard&query=Persoonia&sortorder=epbc_status.

[21] Andres S.E., Powell J.R., Emery N.J., Rymer P.D., Gallagher R.V. (2021). Does threatened species listing status predict climate change risk? A case study with Australian *Persoonia* (*Proteaceae*) species. Glob. Ecol. Conserv..

[22] Corbyn L. (2006). *Persoonia nutans* R. Br (Nodding Geebung) Recovery Plan. https://www.environment.nsw.gov.au/-/media/OEH/Corporate-Site/Documents/Animals-and-plants/Recovery-plans/nodding-geebung-persoonia-nutans-r-br-recovery-plan.pdf.

[23] ANBG Aboriginal Plant Use. https://www.anbg.gov.au/gardens/visiting/exploring/aboriginal-trail/index.html.

[24] Atkinson N. (1949). Antibotics in Australian plants and fungi. Med. J. Aust..

[25] MacLeod J.K., Rasmussen H.B., Willis A.C. (1997). A new glycoside antimicrobial agent from *Persoonia linearia* x *pinifolia*. J. Nat. Prod..

[26] Deans B.J., Kilah N.L., Jordan G.J., Bissember A.C., Smith J.A. (2018). Arbutin Derivatives Isolated from Ancient *Proteaceae*: Potential Phytochemical Markers Present in Bellendena, Cenarrhenes, and Persoonia Genera. J. Nat. Prod..

[27] Cleland J.B. (1950). The naturalist in medicine with particular reference to Australia. Med. J. Aust..

[28] Webb L.J. (1969). The Use of Plant Medicines and Poisons by Australian Aborigines. Mankind.

[29] Jones P.J. (2002). Clinical nutrition: 7. Functional foods—More than just nutrition. CMAJ.

[30] Weston P., Crisp M. (1996). Trans-Pacific biogeographic patterns in the *Proteaceae*. The Origin and Evolution of Pacific Island Biotas, New Guinea to Eastern Polynesia: Patterns and Processes.

[31] Johnson L.A.S., Briggs B.G. (2008). On the *Proteaceae*—The evolution and classification of a southern family. Bot. J. Linn. Soc..

[32] Holmes G.D., Weston P.H., Murphy D.J., Connelly C., Cantrill D.J. (2018). The genealogy of geebungs: Phylogenetic analysis of *Persoonia* (*Proteaceae*) and related genera in subfamily Persoonioideae. Aust. Syst. Bot..

[33] Bernhardt P., Weston P. (1996). The pollination ecology of *Persoonia* (*Proteaceae*) in eastern Australia. Telopea.

[34] AVH Persoonia. https://avh.ala.org.au/occurrences/search?taxa=Persoonia.

[35] USDA (2020). Tree Nuts Annual. https://apps.fas.usda.gov/newgainapi/api/Report/DownloadReportByFileName?fileName=Tree%20Nuts%20Annual_New%20Delhi_India_09-15-2020.

[36] Insanu M., Hartati R., Bajri F., Fidrianny I. (2021). Macadamia Genus: An Updated Review of Phytochemical Compounds and Pharmacological Activities. Biointerface Res. Appl. Chem..

[37] Carrillo W., Carpio C., Morales D., Vilcacundo E., Alvarez M. (2017). Fatty acids composition in macadamia seed oil (*Macadamia integrifolia*) from Ecuador. Asian J. Pharm. Clin. Res..

[38] Vinueza D., Yanza K., Tacchini M., Grandini A., Sacchetti G., Chiurato M.A., Guerrini A. (2018). Flavonoids in Ecuadorian *Oreocallis grandiflora* (Lam.) R. Br.: Perspectives of Use of This Species as a Food Supplement. Evid.-Based Complement. Altern. Med..

[39] Rozas-Muñoz E., Gamé D., Mir-Bonafé J.-F., Piquero-Casals J. (2022). Plant Contact Dermatitis in 2021. Curr. Treat. Options Allergy.

[40] Chia K.A., Koch J.M., Sadler R., Turner S.R. (2015). Developmental phenology of *Persoonia longifolia* (*Proteaceae*) and the impact of fire on these events. Aust. J. Bot..

[41] Criley R.A. (2000). *Proteaceae*: Beyond the big three. Acta Hortic..

[42] Wightman G.M., Jackson D.M., Williams L.L.V. (1991). Alawa Ethnobotany: Aboriginal Plant Use from Minyerri, Northern Australia.

[43] Florin S.A., Fairbairn A.S., Nango M., Djandjomerr D., Marwick B., Fullagar R., Smith M., Wallis L.A., Clarkson C. (2020). The first Australian plant foods at Madjedbebe, 65,000–53,000 years ago. Nat. Commun..

[44] Bauer L.M., Johnston M. (1999). Propagation of Persoonia Virgarta for the Development of a New Floricultural Export Crop.

[45] Stewart K., Percival B. (1997). Bush Foods of New South Wales: A Botanic Record and an Aboriginal Oral History.

[46] Renwick C. (2000). Geebungs and Snake Whistles: Koori People and Plants of Wreck Bay.

[47] Robinson L. (2003). Field Guide to the Native Plants of Sydney.

[48] Edwards C.A., Havlik J., Cong W., Mullen W., Preston T., Morrison D.J., Combet E. (2017). Polyphenols and health: Interactions between fibre, plant polyphenols and the gut microbiota. Nutr. Bull..

[49] Isaacs J. (1987). Bush Food: Aboriginal Food.

[50] ACNT (1988). Traditional Bush Medicines: An Aboriginal Pharmacopoeia.

[51] Turbet P. (1989). The Aborigines of the Sydney District before 1788.

[52] Nash D. (2004). Aboriginal Plant Use in South-Eastern Australia.

[53] BGPA Grevillea Eriostachya. https://www.bgpa.wa.gov.au/about-us/information/our-plants/plants-in-focus/grevillea-eriostachya.

[54] Hansen V., Horsfall J. (2016). Noongar Bush Medicine: Medicinal Plants of the South-West of Western Australia.

[55] Wrigley J.W., Fagg M. (1989). Banksias, Waratahs & Grevilleas, and All Other Plants in the Australian Proteaceae Family.

[56] Cock I.E. (2011). Medicinal and aromatic plants—Australia. Ethnopharmacology, Encyclopedia of Life Support Systems.

[57] Yalo M., Makhaba M., Hussein A.A., Sharma R., Koki M., Nako N., Mabusela W.T. (2022). Characterization of Four New Compounds from Protea cynaroides Leaves and Their Tyrosinase Inhibitory Potential. Plants.

[58] Erazo S., García R., Backhouse N., Lemus I.I., Delporte C., Andrade C. (1997). Phytochemical and biological study of Radal *Lomatia hirsuta* (*Proteaceae*). J. Ethnopharmacol..

[59] Setzer M.C. (2000). Green Gold from Down Under: Bioprospecting for Phytopharmaceuticals from Paluma, North Queensland, Australia. Master’s Thesis.

[60] Twilley D., Lall N., Kuete V. (2014). 16—African Plants with Dermatological and Ocular Relevance. Toxicological Survey of African Medicinal Plants.

[61] Chinsembu K.C., Syakalima M., Semenya S.S. (2019). Ethnomedicinal plants used by traditional healers in the management of HIV/AIDS opportunistic diseases in Lusaka, Zambia. South Afr. J. Bot..

[62] Tlau L., Lalawmpuii L. (2020). Commonly used medicinal plants in N. Mualcheng, Mizoram, India. Sci. Vis..

[63] Ray D.S., Saini M.K. (2022). Impending threats to the plants with medicinal value in the Eastern Himalayas Region: An analysis on the alternatives to its non-availability. Phytomed. Plus.

[64] Palombo E.A., Semple S.J. (2001). Antibacterial activity of traditional Australian medicinal plants. J. Ethnopharmacol..

[65] Xu W.H., Liang Q., Zhang Y.J., Zhao P. (2015). Naturally occurring arbutin derivatives and their bioactivities. Chem. Biodivers..

[66] Rebelo T. (1993). Protea Atlas Project—A spectacular year of atlassing. Veld Flora.

[67] Chia K. (2016). Ecology, Seed Dormancy and Germination Biology of *Persoonia longifolia* for Use in Land Restoration and Horticulture. Ph.D. Thesis.

[68] Seigler D.S. (1998). Plant Secondary Metabolism.

[69] Rodriguez-Casado A. (2016). The Health Potential of Fruits and Vegetables Phytochemicals: Notable Examples. Crit. Rev. Food Sci. Nutr..

[70] Huang Y., Xiao D., Burton-Freeman B.M., Edirisinghe I. (2016). Chemical Changes of Bioactive Phytochemicals during Thermal Processing. Reference Module in Food Science.

[71] Gadea A., Khazem M., Gaslonde T. (2022). Current knowledge on chemistry of *Proteaceae* family, and biological activities of their bis-5-alkylresorcinol derivatives. Phytochem. Rev..

[72] Yang F. (2017). Chemotaxonomy Study of Plants from the Family *Proteaceae* Based on Its Natural Product Profile (Alkaloid). Ph.D. Thesis.

[73] Tang C., Xie B., Sun Z. (2017). Antibacterial activity and mechanism of B-type oligomeric procyanidins from lotus seedpod on enterotoxigenic *Escherichia coli*. J. Funct. Foods.

[74] Patel K., Gadewar M., Tahilyani V., Patel D.K. (2013). A review on pharmacological and analytical aspects of diosmetin: A concise report. Chin. J. Integr. Med..

[75] Kupeli Akkol E., Genç Y., Karpuz B., Sobarzo-Sánchez E., Capasso R. (2020). Coumarins and Coumarin-Related Compounds in Pharmacotherapy of Cancer. Cancers.

[76] Wright A.D., König G.M., Angerhofer C.K., Greenidge P., Linden A., Desqueyroux-Faúndez R. (1996). Antimalarial Activity: The Search for Marine-Derived Natural Products with Selective Antimalarial Activity. J. Nat. Prod..

[77] Ooshiro A., Hiradate S., Kawano S., Takushi T., Fujii Y., Natsume M., Abe H. (2009). Identification and activity of ethyl gallate as an antimicrobial compound produced by *Geranium carolinianum*. Weed Biol. Manag..

[78] Tareq S.M., Islam M., Shahadat S., Guha B., Azad M., Ikram M., Royhan M., Paul A., Kabir M. (2016). Anticancer potential of isolated phytochemicals from grevillea robustra against breast cancer: In slico molecular docking approach. World J. Pharm. Res..

[79] Ryu B., Park E.-J., Doan T.-P., Cho H.-M., An J.-P., Pham T.-L.-G., Pham H.-T.-T., Oh W.-K. (2022). Heliciopsides A–E, Unusual Macrocyclic and Phenolic Glycosides from the Leaves of Heliciopsis terminalis and Their Stimulation of Glucose Uptake. Pharmaceuticals.

[80] Saechan C., Nguyen U.H., Wang Z., Sugimoto S., Yamano Y., Matsunami K., Otsuka H., Phan G.M., Pham V.H., Tipmanee V. (2021). Potency of bisresorcinol from Heliciopsis terminalis on skin aging: In vitro bioactivities and molecular interactions. PeerJ.

[81] Younis M.M., Ayoub I.M., Mostafa N.M., El Hassab M.A., Eldehna W.M., Al-Rashood S.T., Eldahshan O.A. (2022). GC/MS Profiling, Anti-Collagenase, Anti-Elastase, Anti-Tyrosinase and Anti-Hyaluronidase Activities of a *Stenocarpus sinuatus* Leaves Extract. Plants.

[82] Hawary S.S., Abubaker M., Abd El-Kader E.M., Mahrous E.A. (2022). Phytochemical constituents and anti-tyrosinase activity of *Macadamia integrifolia* leaves extract. Nat. Prod. Res..

[83] Ceccarelli S., Grando S., Maatougui M., Michael M., Slash M., Haghparast R., Rahmanian M., Taheri A., Al-Yassin A., Benbelkacem A. (2010). Plant breeding and climate changes. J. Agric. Sci..

[84] Masike K., de Villiers A., Hoffman E.W., Brand D.J., Causon T., Stander M.A. (2020). Detailed Phenolic Characterization of Protea Pure and Hybrid Cultivars by Liquid Chromatography–Ion Mobility–High Resolution Mass Spectrometry (LC-IM-HR-MS). J. Agric. Food Chem..

[85] Ahmed A., Makboul M.A. (2021). Chemical investigation of *Grevillea robusta* A.Cunn flowers. Sphinx J. Pharm. Med. Sci..

[86] Medina J.C., Suárez A.I., Cumbicus N., Morocho V. (2018). Estudio fitoquímico de roupala montana aubl. de la provincia de Loja. Axioma.

[87] Boeing H., Bechthold A., Bub A., Ellinger S., Haller D., Kroke A., Leschik-Bonnet E., Müller M.J., Oberritter H., Schulze M. (2012). Critical review: Vegetables and fruit in the prevention of chronic diseases. Eur. J. Nutr..

[88] Lampe J.W. (1999). Health effects of vegetables and fruit: Assessing mechanisms of action in human experimental studies. Am. J. Clin. Nutr..

[89] Konczak I., Roulle P. (2011). Nutritional properties of commercially grown native Australian fruits: Lipophilic antioxidants and minerals. Food Res. Int..

[90] Salvin S., Bourke M., Byrne T. (2004). The New Crop Industry Handbook.

[91] Dissanayake I.H., Zak V., Kaur K., Jaye K., Ayati Z., Chang D., Li C.G., Bhuyan D.J. (2022). Australian native fruits and vegetables: Chemical composition, nutritional profile, bioactivity and potential valorization by industries. Critical Reviews In Food Science and Nutrition.

[92] Munteanu I.G., Apetrei C. (2021). Analytical Methods Used in Determining Antioxidant Activity: A Review. Int. J. Mol. Sci..

[93] Prior R.L., Wu X., Schaich K. (2005). Standardized methods for the determination of antioxidant capacity and phenolics in foods and dietary supplements. J. Agric. Food Chem..

[94] Chaves N., Santiago A., Alías J.C. (2020). Quantification of the Antioxidant Activity of Plant Extracts: Analysis of Sensitivity and Hierarchization Based on the Method Used. Antioxidants.

[95] Vinueza D., Cajamarca D., Acosta K., Pilco G. (2018). *Oreocallis grandiflora* photoprotective effect against ultraviolet B radiation-induced cell death. Asian J. Pharm. Clin. Res..

[96] Vinueza D., Allauca A.A., Bonilla G.A., León K.L., López S.P. (2018). Assessment of the diuretic and urinary electrolyte effects of hydroalcoholic extract *of Oreocallis grandiflora* (Lam.) R. Br. in Wistar albino rats. Pharmacologyonline.

[97] Kedare S.B., Singh R.P. (2011). Genesis and development of DPPH method of antioxidant assay. J. Food Sci. Technol..

[98] Giang P.M., Thao D.T., Nga N.T., Van Trung B., Anh D.H., Viet P.H. (2019). Evaluation of the Antioxidant, Hepatoprotective, and Anti-Inflammatory Activities of Bisresorcinol Isolated from the Trunk of Heliciopsis Terminalis. Pharm. Chem. J..

[99] Pino Ramos L.L., Jiménez-Aspee F., Theoduloz C., Burgos-Edwards A., Domínguez-Perles R., Oger C., Durand T., Gil-Izquierdo Á., Bustamante L., Mardones C. (2019). Phenolic, oxylipin and fatty acid profiles of the *Chilean hazelnut* (*Gevuina avellana*): Antioxidant activity and inhibition of pro-inflammatory and metabolic syndrome-associated enzymes. Food Chem..

[100] Wu X., Beecher G.R., Holden J.M., Haytowitz D.B., Gebhardt S.E., Prior R.L. (2004). Lipophilic and hydrophilic antioxidant capacities of common foods in the United States. J. Agric. Food Chem..

[101] Somwongin S., Sirilun S., Chantawannakul P., Anuchapreeda S., Chaiyana W. (2022). Potential antioxidant, anti-aging enzymes, and anti-tyrosinase properties of Macadamia (*Macadamia integrifolia*) pericarp waste products. Asia-Pac. J. Sci. Technol..

[102] Ayisi F., Mensah C.N., Borquaye L.S. (2021). In Vivo Antiplasmodial Activity and Toxicological Analyses of the Ethanolic Leaf and Twig Extract of Faurea speciosa Welw. J. Parasitol. Res..

[103] León F., Alfayate C., Batista C.V., López A., Rico M., Brouard I. (2014). Phenolic compounds, antioxidant activity and ultrastructural study from Protea hybrid ‘Susara’. Ind. Crops Prod..

[104] Green K.J., Islam M.K., Lawag I., Locher C., Hammer K.A. (2022). Honeys derived from plants of the coastal sandplains of Western Australia: Antibacterial and antioxidant activity, and other characteristics. J. Apic. Res..

[105] Dailey A., Vuong Q.V. (2015). Optimisation of Ultrasonic Conditions as an Advanced Extraction Technique for Recovery of Phenolic Compounds and Antioxidant Activity from Macadamia (*Macadamia tetraphylla*) Skin Waste. Technologies.

[106] Ramos R.F.d.A. (2015). Constituintes Químicos e Atividade Antioxidante de Roupala Paulensis Sleumer (Proteaceae).

[107] Abba J., Ekwumemgbo P., Dallatu Y., Micheal A.M. (2016). Phytochemical Screening, Antioxidant and Antimicrobial Activities of the Stem Extracts of Woolly Bush (*Adenanthos sericeus*). J. Appl. Sci. Environ. Manag..

[108] Queirós C.S.G.P., Cardoso S., Ferreira J., Miranda I., Lourenço M.J.V., Pereira H. (2020). Characterization of *Hakea sericea* Fruits Regarding Chemical Composition and Extract Properties. Waste Biomass Valorization.

[109] Canha M.N. (2014). Antimicrobial and Anti-Inflammatory Effect of Southern African Plants Against *Propionibacterium acnes*. Master’s Thesis.

[110] Lawal F., Bapela M.J., Adebayo S.A., Nkadimeng S.M., Yusuf A.A., Malterud K.E., McGaw L.J., Tshikalange T.E. (2019). Anti-inflammatory potential of South African medicinal plants used for the treatment of sexually transmitted infections. South Afr. J. Bot..

[111] Leyton M., Mellado M., Jara C., Montenegro I., González S., Madrid A. (2015). Free radical-scavenging activity of sequential leaf extracts of *Embothrium coccineum*. Open Life Sci..

[112] Luís Â., Domingues F., Duarte A. (2011). Bioactive Compounds, RP-HPLC Analysis of Phenolics, and Antioxidant Activity of Some Portuguese Shrub Species Extracts. Nat. Prod. Commun..

[113] Sakulnarmrat K., Srzednicki G., Konczak I. (2014). Composition and inhibitory activities towards digestive enzymes of polyphenolic-rich fractions of Davidson’s plum and quandong. LWT Food Sci. Technol..

[114] Sultanbawa Y., Williams D., Chaliha M., Konczak I., Smyth H. (2015). Changes in Quality and Bioactivity of Native Food during Storage.

[115] Ali A., Cottrell J.J., Dunshea F.R. (2022). Identification and characterization of anthocyanins and non-anthocyanin phenolics from Australian native fruits and their antioxidant, antidiabetic, and anti-Alzheimer potential. Food Res. Int..

[116] Williams D.J., Edwards D., Pun S., Chaliha M., Sultanbawa Y. (2014). Profiling ellagic acid content: The importance of form and ascorbic acid levels. Food Res. Int..

[117] Phan A.D.T., Zhang J., Seididamyeh M., Srivarathan S., Netzel M.E., Sivakumar D., Sultanbawa Y. (2022). Hydrolysable tannins, physicochemical properties, and antioxidant property of wild-harvested *Terminalia ferdinandiana* (exell) fruit at different maturity stages. Front. Nutr..

[118] Cherikoff V., Cherikoff V., Konczak I. (2015). Wild Foods. An Overview of the Health Attributes of Wild Foods.

[119] Lim V., Gorji S.G., Daygon V.D., Fitzgerald M. (2020). Untargeted and Targeted Metabolomic Profiling of Australian Indigenous Fruits. Metabolites.

[120] Konczak I., Zabaras D., Dunstan M., Aguas P., Roulfe P., Pavan A. (2009). Health Benefits of Australian Native Foods—An Evaluation of Health-Enhancing Compounds.

[121] Ali A., Cottrell J.J., Dunshea F.R. (2023). Characterization, Antioxidant Potential, and Pharmacokinetics Properties of Phenolic Compounds from Native Australian Herbs and Fruits. Plants.

[122] Mani J., Johnson J., Hosking H., Hoyos B.E., Walsh K.B., Neilsen P., Naiker M. (2022). Bioassay Guided Fractionation Protocol for Determining Novel Active Compounds in Selected Australian Flora. Plants.

[123] Tan A.C., Konczak I., Ramzan I., Zabaras D., Sze D.M.Y. (2011). Potential Antioxidant, Antiinflammatory, and Proapoptotic Anticancer Activities of *Kakadu Plum* and *Illawarra Plum* Polyphenolic Fractions. Nutr. Cancer.

[124] Akter S., Netzel M.E., Tinggi U., Osborne S.A., Fletcher M.T., Sultanbawa Y. (2019). Antioxidant Rich Extracts of *Terminalia ferdinandiana* Inhibit the Growth of Foodborne Bacteria. Foods.

[125] Tan A.C., Konczak I., Ramzan I., Sze D.M.Y. (2011). Antioxidant and cytoprotective activities of native Australian fruit polyphenols. Food Res. Int..

[126] NSW Food Authority (2021). NSW Government Food Safety Strategy 2015–2021.

[127] Zhang J., Phan A.D.T., Srivarathan S., Akter S., Sultanbawa Y., Cozzolino D. (2022). Proximate composition, functional and antimicrobial properties of wild harvest Terminalia carpentariae fruit. J. Food Meas. Charact..

[128] Courtney R., Sirdaarta J., Matthews B., Cock I.E. (2015). Tannin components and inhibitory activity of *Kakadu plum* leaf extracts against microbial triggers of autoimmune inflammatory diseases. Pharmacogn. J..

[129] Wigmore S., Naiker M., Bean D. (2016). Antimicrobial Activity of Extracts from Native Plants of Temperate Australia. Pharmacogn. Commun..

[130] Boyer H., Cock I.E. (2013). Evaluation of the potential of *Macadamia integriflora* extracts as antibacterial food agents. Pharmacogn. Commun..

[131] Luís Â., Cruz C., Duarte A.P., Domingues F. (2013). An Alkenylresorcinol Derivative from *Hakea sericea* Fruits and their Antimicrobial Activity. Nat. Prod. Commun..

[132] Balouiri M., Sadiki M., Ibnsouda S.K. (2016). Methods for in vitro evaluating antimicrobial activity: A review. J. Pharm. Anal..

[133] Veiga A., Toledo M.d.G.T., Rossa L.S., Mengarda M., Stofella N.C.F., Oliveira L.J., Gonçalves A.G., Murakami F.S. (2019). Colorimetric microdilution assay: Validation of a standard method for determination of MIC, IC50%, and IC90% of antimicrobial compounds. J. Microbiol. Methods.

[134] Elshikh M., Ahmed S., Funston S., Dunlop P., McGaw M., Marchant R., Banat I.M. (2016). Resazurin-based 96-well plate microdilution method for the determination of minimum inhibitory concentration of biosurfactants. Biotechnol. Lett..

[135] Razafintsalama V., Sarter S., Mambu L., Randrianarivo R., Petit T., Rajaonarison J.F., Mertz C., Rakoto D., Jeannoda V. (2013). Antimicrobial activities of *Dilobeia thouarsii* Roemer and Schulte, a traditional medicinal plant from Madagascar. South Afr. J. Bot..

[136] Canales N., Montenegro I., Párraga M., Olguín Y., Godoy P., Werner E., Madrid A. (2016). In Vitro Antimicrobial Activity of *Embothrium coccineum* Used as Traditional Medicine in Patagonia against Multiresistant Bacteria. Molecules.

[137] Setzer M.C., Schmidt J.M., Irvine A.K., Jackes B., Setzer W. (2006). Biological activity of rainforest plant extracts from Far North Queensland, Australia. Aust. J. Med. Herbal.

[138] Violante I.M., Hamerski L., Garcez W.S., Batista A.L., Chang M.R., Pott V.J., Garcez F.R. (2012). Antimicrobial activity of some medicinal plants from the cerrado of the centralwestern region of Brazil. Braz. J. Microbiol..

[139] Mølgaard P., Holler J.G., Asar B., Liberna I., Rosenbæk L.B., Jebjerg C.P., Jørgensen L., Lauritzen J., Guzman A., Adsersen A. (2011). Antimicrobial evaluation of Huilliche plant medicine used to treat wounds. J. Ethnopharmacol..

[140] Madureira A.M., Duarte A., Teixeira G. (2012). Antimicrobial activity of selected extracts from *Hakea salicifolia* and *H. sericeae* (*Proteaceae*) against Staphylococcus aureus multiresistant strains. South Afr. J. Bot..

[141] Cock I.E., Winnett V., Sirdaarta J., Matthews B. (2015). The potential of selected Australian medicinal plants with anti-Proteus activity for the treatment and prevention of rheumatoid arthritis. Pharmacogn. Mag..

[142] Qongqo A., Nchu F., Geerts S. (2022). Relationship of alien species continues in a foreign land: The case of *Phytophthora* and Australian *Banksia* (*Proteaceae*) in South African Fynbos. Ecol. Evol..

[143] Setzer M.C., Setzer W.N., Jackes B.R., Gentry G.A., Moriarity D.M. (2001). The Medicinal Value of Tropical Rainforest Plants from Paluma, North Queensland, Australia. Pharm. Biol..

[144] Razafintsalama V.E., Ralambonirina Rasoarivelo S.T., Randriamialinoro F., Ranarivelo L., Rakotonandrasana S.R., Petit T., Sarter S. (2017). Antibacterial activities of fourteen medicinal plants from the endemic plant diversity of Madagascar. South Afr. J. Bot..

[145] Brady N., Molan P., Bang L. (2004). A survey of non-manuka New Zealand honeys for antibacterial and antifungal activities. J. Apic. Res..

[146] Simonsen H.T., Adsersen A., Berthelsen L., Christensen S.B., Guzmán A., Mølgaard P. (2006). Ethnopharmacological evaluation of radal (leaves of *Lomatia hirsuta*) and isolation of 2-methoxyjuglone. BMC Complement. Med. Ther..

[147] Bussmann R.W., Malca-García G., Glenn A., Sharon D., Chait G., Díaz D., Pourmand K., Jonat B., Somogy S., Guardado G. (2010). Minimum inhibitory concentrations of medicinal plants used in Northern Peru as antibacterial remedies. J. Ethnopharmacol..

[148] Suffredini I., Paciencia M., Varella A., Younes R. (2007). Antibacterial activity of Brazilian Amazon plant extracts. Braz. J. Infect. Dis..

[149] Cock I. (2008). Antibacterial and Antifungal Activity of Buckinghamia Celsissima Leaf Extracts. Internet J. Microbiol..

[150] Chang J., Inui T. (2005). Novel Phenolic Glycoside Dimer and Trimer from the Whole Herb of Pyrola rotundifolia. Chem. Pharm. Bull..

[151] Perry N.B., Brennan N.J. (1997). Antimicrobial and cytotoxic phenolic glycoside esters from the New Zealand tree *Toronia toru*. J. Nat. Prod..

[152] Marcus J.P., Goulter K.C., Green J.L., Harrison S.J., Manners J.M. (1997). Purification, Characterisation and cDNA Cloning of an Antimicrobial Peptide from *Macadamia integrifolia*. Eur. J. Biochem..

[153] Marcus J.P., Green J.L., Goulter K.C., Manners J.M. (1999). A family of antimicrobial peptides is produced by processing of a 7S globulin protein in *Macadamia integrifolia* kernels. Plant J..

[154] Castillo U., Harper J.K., Strobel G.A., Sears J., Alesi K., Ford E., Lin J., Hunter M., Maranta M., Ge H. (2003). Kakadumycins, novel antibiotics from *Streptomyces* sp. NRRL 30566, an endophyte of *Grevillea pteridifolia*. FEMS Microbiol. Lett..

[155] Razafintsalama V., Girardot M., Randrianarivo R., Rakoto D., Sarter S., Petit T., Ralambonirina S., Deville A., Grellier P., Jeannoda V. (2013). Dilobenol A–G, Diprenylated Dihydroflavonols from the Leaves of *Dilobeia thouarsii*. Eur. J. Org. Chem..

[156] Cock I. (2019). *Grevillea juncifolia* Hook. and *Grevillea robusta* A. Cunn. Ex. R. Br. Methanolic Leaf and Flower Extracts Inhibit the Growth of Gram Positive and Gram Negative Bacteria. Pharmacogn. Commun..

[157] Almeida R., Silva O., Candido E., Moreira J., Jojoa D., Gomes D., Freire M., Burgel P., Oliveira Júnior N., Arboleda Valencia J. (2014). Screening and isolation of antibacterial proteinaceous compounds from flower tissues: Alternatives for treatment of healthcare-associated infections. Tang Humanit. Med..

[158] Holler J.G., Søndergaard K., Slotved H.C., Gúzman A., Mølgaard P. (2012). Evaluation of the antibacterial activity of Chilean plants traditionally used for wound healing therapy against multidrug-resistant *Staphylococcus aureus*. Planta Medica.

[159] Gasa N. (2015). Antibiofilm Activity of South African Plant Extracts Against *Mycobacterium* spp. and Their Mechanism of Action Using Mycothiol Reductase. Ph.D. Thesis.

[160] Luís A., Breitenfeld L., Ferreira S., Duarte A.P., Domingues F. (2014). Antimicrobial, antibiofilm and cytotoxic activities of *Hakea sericea* Schrader extracts. Pharmacogn. Mag..

[161] Vambe M., Aremu A.O., Chukwujekwu J.C., Gruz J., Luterová A., Finnie J.F., Van Staden J. (2020). Antibacterial, Mutagenic Properties and Chemical Characterisation of Sugar Bush (*Protea caffra* Meisn.): A South African Native Shrub Species. Plants.

[162] Podschun R., Ullmann U. (1998). Klebsiella spp. as nosocomial pathogens: Epidemiology, taxonomy, typing methods, and pathogenicity factors. Clin. Microbiol. Rev..

[163] Nizet V., Klein J.O., Remington J.S., Klein J.O., Wilson C.B., Nizet V., Maldonado Y.A. (2011). CHAPTER 6—Bacterial Sepsis and Meningitis. Infectious Diseases of the Fetus and Newborn.

[164] Jahani S., Bazi S., Shahi Z., Sheykhzade Asadi M., Mosavi F., Sohil Baigi G. (2017). Antifungal Effect of the Extract of the Plants Against *Candida albicans*. Int. J. Infect. Dis..

[165] Seok H., Huh K., Cho S.Y., Kang C.-I., Chung D.R., Huh W.S., Park J.B., Peck K.R. (2020). Invasive Fungal Diseases in Kidney Transplant Recipients: Risk Factors for Mortality. J. Clin. Med..

[166] Cock I., Maen A. (2015). Inhibitory activity of high antioxidant Australian native fruits against the bacterial triggers of selected autoimmune diseases. Pharmacogn. Commun..

[167] Cheesman M.J., White A., Matthews B., Cock I.E. (2019). *Terminalia ferdinandiana* Fruit and Leaf Extracts Inhibit Methicillin-Resistant *Staphylococcus aureus* Growth. Planta Medica.

[168] Noé W., Murhekar S., White A., Davis C., Cock I.E. (2019). Inhibition of the growth of human dermatophytic pathogens by selected australian and asian plants traditionally used to treat fungal infections. J. Mycol. Médicale.

[169] WHO Cancer Over Time. https://gco.iarc.fr/overtime/en.

[170] Solowey E., Lichtenstein M., Sallon S., Paavilainen H., Solowey E., Lorberboum-Galski H. (2014). Evaluating Medicinal Plants for Anticancer Activity. Sci. World J..

[171] Larramendy M., Soloneski S. (2018). Genotoxicity: A Predictable Risk to Our Actual World.

[172] Wang P., Henning S.M., Heber D. (2010). Limitations of MTT and MTS-Based Assays for Measurement of Antiproliferative Activity of Green Tea Polyphenols. PLoS ONE.

[173] Deng J.Z., Starck S.R., Sun D.A., Sabat M., Hecht S.M. (2000). A new 7,8-euphadien-type triterpenoid from Brackenridgea nitida and Bleasdalea bleasdalei that inhibits DNA polymerase beta. J. Nat. Prod..

[174] Yu H., Zhang X., Li X., Zeng F., Ruan H. (2013). 2-Methoxyjuglone Induces Apoptosis in HepG2 Human Hepatocellular Carcinoma Cells and Exhibits in Vivo Antitumor Activity in a H22 Mouse Hepatocellular Carcinoma Model. J. Nat. Prod..

[175] Jolly C., Thoison O., Martin M.-T., Dumontet V., Gilbert A., Pfeiffer B., Léonce S., Sévenet T., Guéritte F., Litaudon M. (2008). Cytotoxic turrianes of Kermadecia elliptica from the New Caledonian rainforest. Phytochemistry.

[176] Qi W., Ou N., Wu X., Xu H. (2016). New arbutin derivatives from the leaves of Heliciopsis lobata with cytotoxicity. Chin. J. Nat. Med..

[177] Kamagaju L., Morandini R., Bizuru E., Nyetera P., Nduwayezu J.B., Stévigny C., Ghanem G., Duez P. (2013). Tyrosinase modulation by five Rwandese herbal medicines traditionally used for skin treatment. J. Ethnopharmacol..

[178] Ebrahim H., Osman S., Haffez H., Hassan Z. (2020). In-vitro screening of some plant extracts for their potential anticancer activity. Afr. J. Tradit. Complement. Altern. Med..

[179] Fiorito S., Epifano F., Bruyère C., Mathieu V., Kiss R., Genovese S. (2014). Growth inhibitory activity for cancer cell lines of lapachol and its natural and semi-synthetic derivatives. Bioorg. Med. Chem. Lett..

[180] Chuang T.-H., Chan H.-H., Wu T.-S., Li C.-F. (2011). Chemical Constituents and Biological Studies of the Leaves of *Grevillea robusta*. Molecules.

[181] Khazem M., Gaslonde T., Dumontet V., Poullain C., Litaudon M., Michel S. (2014). Cytotoxic turrianes from Kermadecia elliptica: Hemisynthesis and biological activities of kermadecin A derivatives. Phytochem. Lett..

[182] Chuang T., Wu P. (2007). Cytotoxic 5-Alkylresorcinol Metabolites from the Leaves of *Grevillea robusta*. J. Nat. Prod..

[183] Ullah M., Sikder M.A.A., Sharmin T., Rashid M. (2014). Pharmacological Activities of *Grevillea robusta*, a Medicinal Plant of Bangladesh. Bangladesh Phramaceutical J..

[184] Cantrell C.L., Berhow M.A., Phillips B.S., Duval S.M., Weisleder D., Vaughn S.F. (2003). Bioactive crude plant seed extracts from the NCAUR oilseed repository. Phytomedicine.

[185] Akhtar M.A. (2018). Australian Native Plants—A Source of Novel Anti-Inflammatory Compounds. Ph.D. Thesis.

[186] Howlader N., Noone A.M., Krapcho M., Miller D., Brest A., Yu M., Ruhl J., Tatalovich Z., Mariotto A., Lewis D.R. (2021). SEER Cancer Statistics Review, 1975–2018.

[187] Lang G., Cole A.L.J., Blunt J.W., Robinson W.T., Munro M.H.G. (2007). Excelsione, a Depsidone from an Endophytic Fungus Isolated from the New Zealand Endemic Tree Knightia excelsa. J. Nat. Prod..

[188] Yu H.-y., Liu L., Li J., Liu D., Ruan H.-l. (2022). 2-Methoxyjuglone, a Promising Bioactive Compound for Pharmaceutical and Agricultural Purposes: A Review. Curr. Med. Sci..

[189] Brotz E., Herrmann J., Wiese J., Zinecker H., Maier A., Kelter G., Imhoff J.F., Müller R., Paululat T. (2014). Synthesis and Cytotoxic Activity of a Small Naphthoquinone Library: First Synthesis of Juglonbutin. Eur. J. Org. Chem..

[190] Fási L., Di Meo F., Kuo C.-Y., Stojkovic Buric S., Martins A., Kúsz N., Béni Z., Dékány M., Balogh G.T., Pesic M. (2019). Antioxidant-Inspired Drug Discovery: Antitumor Metabolite Is Formed in Situ from a Hydroxycinnamic Acid Derivative upon Free-Radical Scavenging. J. Med. Chem..

[191] Shabbir M., Ziauddin Sultani S., Jabbar A., Iqbal Choudhary M. (1997). Cinnamates and coumarins from the leaves of Murraya paniculata. Phytochemistry.

[192] Ng M.K., Abdulhadi-Noaman Y., Cheah Y.K., Yeap S.K., Alitheen N. (2012). Bioactivity studies and chemical constituents of *Murraya paniculata* (Linn) Jack. Int. Food Res. J..

[193] Whysner J., Verna L., English J.C., Williams G.M. (1995). Analysis of Studies Related to Tumorigenicity Induced by Hydroquinone. Regul. Toxicol. Pharmacol..

[194] Bertanha C.S., Januário A.H., Alvarenga T.A., Pimenta L.P., Silva M.L.A.e., Cunha W.R., Pauletti P.M. (2014). Quinone and Hydroquinone Metabolites from the Ascidians of the Genus Aplidium. Mar. Drugs.

[195] Sunassee S., Davies-Coleman M. (2012). Cytotoxic and antioxidant marine prenylated quinones and hydroquinones. Nat. Prod. Rep..

[196] Herrmann F.C., Lenz M., Jose J., Kaiser M., Brun R., Schmidt T.J. (2015). In Silico Identification and in Vitro Activity of Novel Natural Inhibitors of Trypanosoma brucei Glyceraldehyde-3-phosphate-dehydrogenase. Molecules.

[197] Beg S., Swain S., Hasan H., Barkat M.A., Hussain M.S. (2011). Systematic review of herbals as potential anti-inflammatory agents: Recent advances, current clinical status and future perspectives. Pharmacogn. Rev..

[198] Dvorakova M., Landa P. (2017). Anti-inflammatory activity of natural stilbenoids: A review. Pharmacol. Res..

[199] Akhtar M.A., Raju R., Beattie K.D., Bodkin F., Münch G. (2016). Medicinal Plants of the Australian Aboriginal Dharawal People Exhibiting Anti-Inflammatory Activity. Evid.-Based Complement. Altern. Med..

[200] Oguntibeju O.O. (2018). Medicinal plants with anti-inflammatory activities from selected countries and regions of Africa. J. Inflamm. Res..

[201] Hongzhi D., Xiaoying H., Yujie G., Le C., Yuhuan M., Dahui L., Luqi H. (2022). Classic mechanisms and experimental models for the anti-inflammatory effect of traditional Chinese medicine. Anim. Models Exp. Med..

[202] Fawole O.A., Ndhlala A.R., Amoo S.O., Finnie J.F., Van Staden J. (2009). Anti-inflammatory and phytochemical properties of twelve medicinal plants used for treating gastro-intestinal ailments in South Africa. J. Ethnopharmacol..

[203] Alvarez M.E., Rotelli A.E., Pelzer L.E., Saad J.R., Giordano O. (2000). Phytochemical study and anti-inflammatory properties of Lampaya hieronymi Schum. ex Moldenke. Il Farmaco.

[204] Wang H., Leach D.N., Thomas M.C., Blanksby S.J., Forster P.I., Waterman P.G. (2011). Bisresorcinol Derivatives from Grevillea glauca. Helv. Chim. Acta.

[205] Chen Y.-P. (1980). Recent developments of natural product chemistry in Taiwan. Korean J. Pharmacogn..

[206] Kashyap D., Sharma A., Tuli H.S., Punia S., Sharma A.K. (2016). Ursolic Acid and Oleanolic Acid: Pentacyclic Terpenoids with Promising Anti-Inflammatory Activities. Recent Pat. Inflamm. Allergy. Drug. Discov..

[207] Ahmed A.S. (2006). Phytochemical and biological study of *Grevillea robusta* A. Cunn cultivated in Egypt. Bull. Pharm. Sciences. Assiut.

[208] Sweeney A.P., Wyllie S.G., Shalliker R.A., Markham J.L. (2001). Xanthine oxidase inhibitory activity of selected Australian native plants. J. Ethnopharmacol..

[209] Meloncelli D. (2019). Authentication of Australian and New Zealand Honey Origins by Chromatography, and Their Anti-Inflammatory Properties. Ph.D. Thesis.

[210] Sangphum A. (2016). Biological Activities of Thai Traditional Remedy Called Prasachangdaeng and Its Plant Ingredients. Ph.D. Thesis.

[211] Denaro M., Smeriglio A., Barreca D., De Francesco C., Occhiuto C., Milano G., Trombetta D. (2020). Antiviral activity of plants and their isolated bioactive compounds: An update. Phytother. Res..

[212] Decosterd L.A., Parsons I.C., Gustafson K.R., Cardellina J.H., McMahon J.B., Cragg G.M., Murata Y., Pannell L.K., Steiner J.R. (1993). HIV inhibitory natural products. 11. Structure, absolute stereochemistry, and synthesis of conocurvone, a potent, novel HIV-inhibitory naphthoquinone trimer from a *Conospermum* sp. J. Am. Chem. Soc..

[213] Dhar M., Dhawan B.N., Prasad C., Rastogi R., Singh K., Tandon J. (1974). Screening of Indian plants for biological activity: Part V. Indian J. Exp. Biol..

[214] Wink M. (2012). Medicinal plants: A source of anti-parasitic secondary metabolites. Molecules.

[215] Cunha N.L., Uchôa C.J.d.M., Cintra L.S., Souza H.C.d., Peixoto J.A., Silva C.P., Magalhães L.G., Gimenez V.M.M., Groppo M., Rodrigues V. (2012). In Vitro Schistosomicidal Activity of Some Brazilian Cerrado Species and Their Isolated Compounds. Evid.-Based Complement. Altern. Med..

[216] Takahashi M., Fuchino H., Satake M., Agatsuma Y., Sekita S. (2004). In Vitro Screening of Leishmanicidal Activity in Myanmar Timber Extracts. Biol. Pharm. Bull..

[217] Aubouy A., Camara A., Haddad M., Chassagne F. (2022). Chapter 8—Medicinal plants from West Africa used as antimalarial agents: An overview. Medicinal Plants as Anti-Infectives.

[218] Laryea M.K., Borquaye L.S. (2019). Antimalarial Efficacy and Toxicological Assessment of Extracts of Some Ghanaian Medicinal Plants. J. Parasitol. Res..

[219] Cock I.E., Selesho M.I., van Vuuren S.F. (2019). A review of the traditional use of southern African medicinal plants for the treatment of malaria. J. Ethnopharmacol..

[220] Yamashita-Higuchi Y., Sugimoto S., Matsunami K., Otsuka H., Nakai T. (2014). Grevillosides J-Q, arbutin derivatives from the leaves of *Grevillea robusta* and their melanogenesis inhibitory activity. Chem. Pharm. Bull..

[221] Sonka L. (2018). Exploring Anti-Tyrosinase Bioactive Compounds from the Cape Flora.

[222] Beniddir M.A., Simonin A.-L., Martin M.-T., Dumontet V., Poullain C., Guéritte F., Litaudon M. (2010). Turrianes from Kermadecia rotundifolia as new acetylcholinesterase inhibitors. Phytochem. Lett..

[223] Roufogalis B.D., Li Q., Tran V.H., Kable E.P.W., Duke C.C. (1999). Investigation of plant-derived phenolic compounds as plasma membrane Ca2+-ATPase inhibitors with potential cardiovascular activity. Drug Dev. Res..

[224] Migas P., Krauze-Baranowska M. (2015). The significance of arbutin and its derivatives in therapy and cosmetics. Phytochem. Lett..

[225] Masuda T., Mizuguchi S., Tanaka T., Iritani K., Takeda Y., Yonemori S. (2000). Isolation and Structure Determination of New Antioxidative Ferulic Acid Glucoside Esters from the Rhizome of Alpinia speciosa, a Zingiberaceae Plant Used in Okinawan Food Culture. J. Agric. Food Chem..

[226] Gendaram O., Choi Y.H., Sun-young K., Ryu S.Y. (2011). Anti-oxidative and antibacterial constituents from *Sedum hybridum*. Nat. Prod. Sci..

[227] Pavlović D.R., Branković S., Kovačević N., Kitić D., Veljković S. (2011). Comparative Study of Spasmolytic Properties, Antioxidant Activity and Phenolic Content of Arbutus unedo from Montenegro and Greece. Phytother. Res..

[228] Gascoigne R.E., White D.E. (1949). A Survey of Anthocyanins in Australian Flora: Issued 23 November 1948. J. Proc. R. Soc. N. S. W..

[229] Lila M.A. (2004). Anthocyanins and Human Health: An In Vitro Investigative Approach. J. Biomed. Biotechnol..

[230] Cornforth J.W. (1938). The glycoside of *Persoonia salicina* fruits. J. Proc. R. Soc. N. S. W..

[231] Cannon J., Metcalf B. (1971). Phenolic constituents of *Persoonia elliptica* (*Proteaceae*). Aust. J. Chem..

[232] Bateman P.W., Pearlman P., Robertson P., Schultz B., Wardell-Johnson G. (2017). Is the Biodiversity Conservation Act 2016 (WA) fit for purpose?. Pac. Conserv. Biol..

[233] Ward M.S., Simmonds J.S., Reside A.E., Watson J.E.M., Rhodes J.R., Possingham H.P., Trezise J., Fletcher R., File L., Taylor M. (2019). Lots of loss with little scrutiny: The attrition of habitat critical for threatened species in Australia. Conserv. Sci. Pract..

